# Triaxial Accelerometry Sensor for Respiratory-Effort Phase Landmark Detection During Sleep: A Cross-Modal Weak Supervision Approach

**DOI:** 10.3390/bioengineering13101205

**Published:** 2026-10-16

**Authors:** Mengshuang Lv, Jianhong Liao, Yunhan Shi, Bowen Zhang, Qiying Zeng, Yanru Li, Demin Han

**Affiliations:** 1 Obstructive Sleep Apnea-Hypopnea Syndrome Clinical Diagnosis and Therapy and Research Centre, Beijing Tongren Hospital, Capital Medical University, Beijing 100005, China; lvmengshuang163@163.com (M.L.); liaojh@ccmu.edu.cn (J.L.); yunhanshi@163.com (Y.S.); zzzhangbw@163.com (B.Z.); qiyingzeng36@gmail.com (Q.Z.); 2 Key Laboratory of Otorhinolaryngology Head and Neck Surgery, Capital Medical University, Ministry of Education, Beijing 100005, China; 3 Key Laboratory of Biopharmaceuticals and Medical Devices, Xinjiang Medical University, Urumqi 830054, China

**Keywords:** triaxial accelerometry sensor, respiratory phase-landmark detection, weak supervision

## Abstract

Reliable respiratory phase sensing is important for respiratory-synchronized hypoglossal nerve stimulation (HGNS), but conventional sensing approaches may impose additional burden. This pilot study investigated whether a surface triaxial accelerometry sensor (TAS) could detect thoracic respiratory inductance plethysmography (RIP) troughs, which were used as operational phase landmarks representing the transition from end expiration to subsequent inspiration, rather than as directly established physiological respiratory onsets. Ten participants underwent synchronized overnight polysomnography and surface TAS recording, providing 5377.3 min of analyzable data and 75,499 automatically generated RIP landmarks. Two complementary Conformer models were trained using Gaussian weak labels and combined using multi-offset dual-conformer fusion. Generalization was evaluated using outer leave-one-subject-out validation, with fusion weights and event thresholds selected from training-side validation data. At a ±1.0 s matching tolerance, the fusion achieved participant-mean precision, recall, and F1 scores of 0.78, 0.82, and 0.80, respectively, with a 95% confidence interval of 0.75–0.85 for the mean F1 score. It outperformed each component model in 9 of 10 participants; however, its F1 gain over otherwise identical single-grid fusion was 0.0036, below the prespecified exploratory advancement margin. These findings provide preliminary evidence that surface TASs can recover an RIP-trough-derived operational phase landmark in unseen same-center participants. Independent multimodal adjudication, external replication, and full-night causal testing are required before clinical or respiratory-synchronized HGNS applications.

## 1. Introduction

Obstructive sleep apnea (OSA) is a common sleep-disordered breathing disorder characterized by recurrent upper-airway obstruction during sleep, intermittent hypoxemia, and sleep fragmentation [1]. Global estimates suggest that OSA affects a large adult population worldwide, with a substantial proportion of participants having moderate-to-severe disease requiring active treatment [2]. For participants with moderate-to-severe OSA who cannot use or tolerate positive airway pressure (PAP), hypoglossal nerve stimulation (HGNS) is an established alternative that recruits upper-airway dilator muscles to maintain pharyngeal patency [3].

Respiratory synchronization is central to contemporary HGNS therapy. Instead of providing fixed frequency stimulation throughout the respiratory cycle, closed-loop HGNS systems monitor respiratory effort and deliver stimulation in coordination with inspiration [4]. Inspiratory negative pressure increases the tendency of the pharyngeal airway to collapse, whereas timely upper-airway muscle activation can help stabilize the airway [5,6].

Reliable detection of inspiratory phase is therefore important for reducing unnecessary stimulation during expiration, avoiding ineffective energy delivery, and limiting excessive neuromuscular loading. However, a useful sensing algorithm must be evaluated against a physiologically defensible target and a clinically justified timing window; event detection within a broad tolerance alone does not establish therapeutic triggering validity.

The currently approved Inspire system (Inspire Medical Systems, Golden Valley, MN) includes an implantable pulse generator, a stimulation lead, and a respiratory sensing lead [7]. The respiratory sensing lead detects respiratory effort through a pressure-sensitive membrane and is implanted in the intercostal region [8]. This configuration has proven clinical value, but it also requires an additional implanted lead and a separate thoracic surgical site. Lead-related issues remain relevant to long-term device reliability. Reported adverse effects of implanted HGNS systems include changes in stimulation over time associated with tissue changes, electrode shift, loose electrical connections, or lead fracture [9,10,11]. These limitations support the need for simpler, less invasive, and mechanically robust sensing strategies for future closed-loop HGNS systems.

Physiological and biomedical information can be captured using electrical, mechanical, pressure, acoustic, optical, and magnetic sensing platforms [12,13]. Illustrating this technological diversity, magnetoimpedance sensors have detected cardiac and smooth-muscle biomagnetic activity [14], magnetomyography has been explored for skeletal-muscle assessment [15], and magnetic nanoparticle labels have extended magnetic sensing to vascular applications [16]. In sleep medicine, tracheal sound and movement sensing can identify respiratory phases [17], while chest-worn accelerometry can recover respiratory patterns and effort during sleep [18,19].

Among these approaches, respiratory effort in clinical sleep studies is commonly assessed using esophageal pressure monitoring or thoracoabdominal respiratory inductance plethysmography (RIP) belts [20,21,22]. Esophageal manometry provides a direct measure of respiratory effort, but its invasiveness makes it unsuitable for routine use or chronic monitoring. RIP belts in polysomnography (PSG) are widely used as non-invasive effort sensors in sleep studies and provide clinically useful information on thoracoabdominal motion and respiratory effort [23]. However, neither esophageal pressure monitoring nor RIP belts are suitable for chronic implantation in HGNS systems.

Miniature triaxial accelerometry sensors (TASs) are attractive candidates for low-burden respiratory motion sensing. Breathing generates subtle periodic movements of the chest wall and surrounding soft tissues, and TASs can capture these movements along three spatial axes while also reflecting posture and body motion [24,25,26,27]. Previous studies have shown that chest-worn TASs can support respiratory monitoring under relatively stable conditions [27,28,29,30]. In sleep medicine, TASs have mainly been used for respiratory rate, respiratory cessation, actigraphy, posture, or gross movement [31,32,33,34]. Landmark extraction during sleep remains challenging because respiratory motion can be masked by movement, posture transitions, sensor displacement, and irregular effort [18,27,28,31,35]. Conventional band-pass, phase, peak-detection, and decomposition methods can degrade when morphology varies [18,27,28,36]. Data-driven models may tolerate more variability [19,37], but manual breath-level annotation across complete nights is costly. However, previous TAS studies have primarily addressed respiratory rate estimation, respiratory-event screening, posture, or respiratory-effort waveform reconstruction. Breath-level identification of a physiologically defined respiratory phase landmark from subtle surface movements during sleep, particularly across individuals, sleep stages, and body positions, remains insufficiently established.

This pilot study aimed to develop and evaluate a cross-modal weak-supervision approach for detecting thoracic RIP troughs from surface triaxial accelerometry signals during sleep. The thoracic RIP trough was defined as an operational respiratory-phase landmark representing the transition from end expiration to subsequent inspiration. The primary objective was to evaluate detection performance in unseen individuals using subject-level leave-one-subject-out validation. Secondary objectives were to compare complementary model and fusion configurations and to descriptively examine performance across sleep stages and body positions.

## 2. Materials and Methods

### 2.1. Study Population

Participants with clinically suspected OSA who underwent overnight PSG were prospectively enrolled in this study. Eligible participants were adults aged 18–70 years who were able to complete overnight PSG monitoring and tolerate placement of the surface TAS. Exclusion criteria were pulmonary or thoracic conditions that could affect chest-wall excursion; sleep disorders other than OSA; and inability to provide informed consent. Demographic and clinical information, including age, sex, ethnicity, body mass index (BMI), PSG results, was collected for each participant. Enrollment was based on the prespecified eligibility criteria rather than demographic stratification; neither sex nor ethnicity was used as an inclusion criterion. The study protocol was approved by the Ethics Committee of Beijing Tongren Hospital, Capital Medical University (Approval No. TRIITKX-2024-183). Written informed consent was obtained from all participants before enrollment.

### 2.2. Data Acquisition and Sensor Configuration

**Figure 1:**
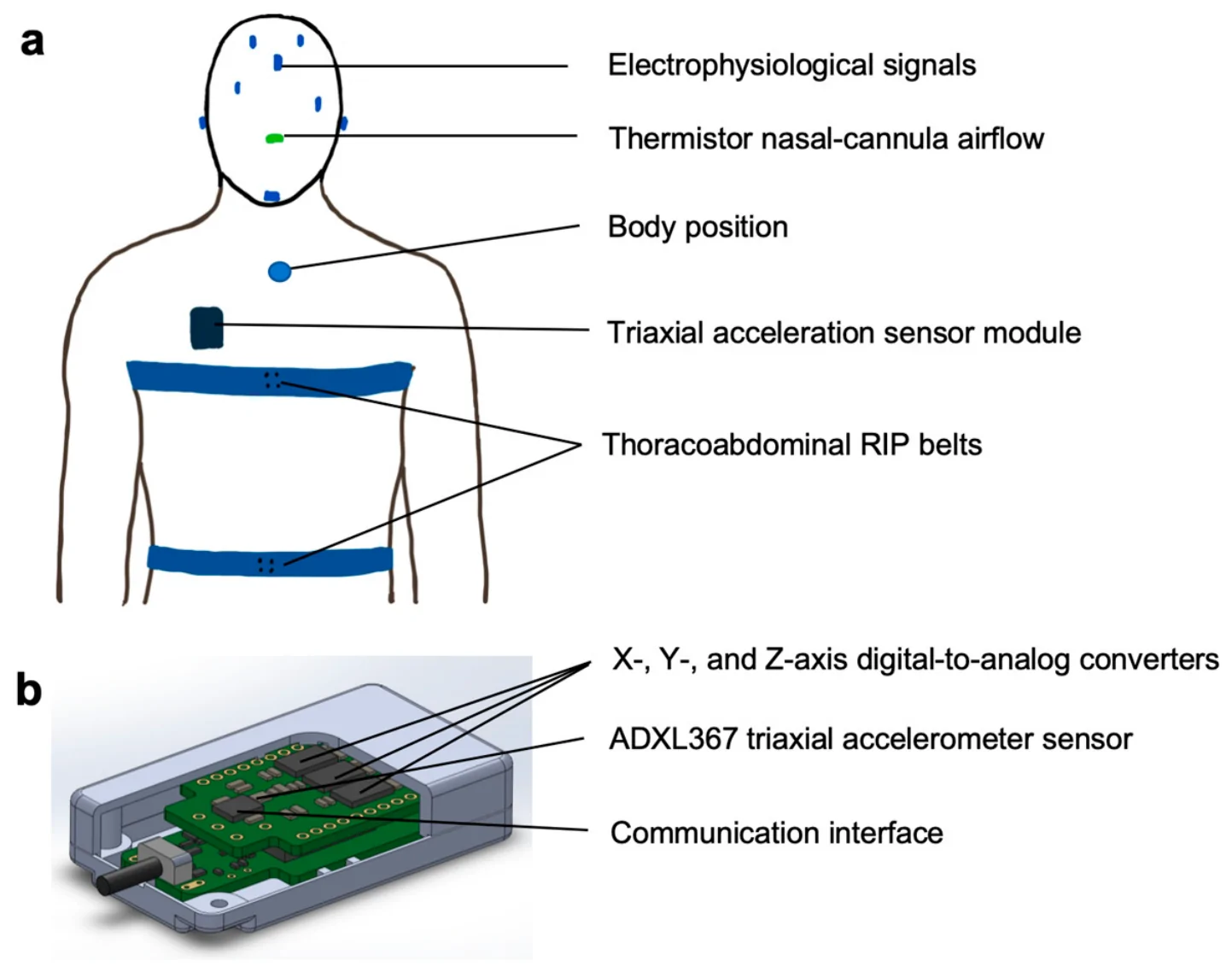
Overnight recording configuration and custom triaxial acceleration sensor module. (**a**) Placement of the polysomnographic sensors, thoracoabdominal respiratory inductance plethysmography belts, and surface triaxial acceleration sensor module over the right parasternal third intercostal space. (**b**) Internal configuration of the custom sensor module, showing the ADXL367 triaxial accelerometer, axis-specific digital-to-analog conversion, and communication interface. RIP, respiratory inductance plethysmography.

All participants underwent overnight PSG recording using the Grael system (Compumedics Inc., Abbotsford, Australia). The recorded signals included electrophysiological signals for sleep staging and sleep event assessment, thermistor and nasal-cannula airflow, thoracic and abdominal RIP belts, oximetry, body position, limb movement, and actigraphy. The PSG recordings were scored by two qualified sleep physicians in accordance with the latest AASM rules (AASM Scoring Manual v3). Simultaneously, a custom triaxial acceleration sensor module (TASM; Amygdala Neuro, Chengdu, China) was placed over the right parasternal third intercostal space to record respiration-related triaxial acceleration signals throughout the overnight study (Figure 1). All recordings were conducted in the same sleep-study room at the Sleep Center of Beijing Tongren Hospital under the same overnight recording conditions. The same Grael system and TASM configuration, including the sensor placement and acquisition settings, were used for all participants.

The custom TASM comprised an ADXL367 triaxial microelectromechanical systems (MEMS) accelerometer (Analog Devices, Wilmington, MA, USA; Figure 1) and a 16-bit RISC MSP430 microcontroller with an onboard 12-bit digital-to-analog converter (DAC) (Texas Instruments, Dallas, TX, USA). The accelerometer was configured with a ±2 g measurement range and a nominal sensitivity of 0.25 mg/LSB. Triaxial acceleration samples were generated at 50 Hz, buffered in the accelerometer’s internal first-in, first-out memory, and retrieved by the microcontroller through an 8 MHz serial peripheral interface. The digital X-, Y-, and *Z*-axis signals were converted by the onboard DAC into separate analog voltage waveforms and transmitted to three analog input channels of the Grael system. The Grael system recorded the three TAS channels at 32 Hz, consistent with the sampling rate of the RIP signals used in this analysis. Recording the TAS and PSG signals through the same acquisition system and on the same computer provided hardware-level temporal alignment. Residual interchannel synchronization error and the calibration between analog voltage and physical acceleration were not independently quantified in the present study.

### 2.3. Physiological Target, Weak-Label Generation, and TAS Preprocessing

#### 2.3.1. Thoracic RIP Belt Signal Preprocessing

The thoracic RIP channel selected for label generation was processed separately for each participant. At the analysis sampling rate of 32 Hz, a 15-sample (0.469 s) median filter was first applied, followed by a second-order Butterworth band-pass design with cutoff frequencies of 0.1 and 0.7 Hz [38]. The Butterworth filter was applied bidirectionally in the forward and reverse directions to obtain zero-phase filtering. The resulting signal was standardized using the mean and standard deviation calculated over the participant-specific record.

#### 2.3.2. Multimodal PSG Validation of the RIP-Derived Reference Landmarks

To assess whether the RIP-trough-derived operational phase landmarks corresponded temporally to expert-annotated respiratory onsets, two sleep experts annotated 100 randomly selected, non-overlapping 60 s epochs, comprising 10 epochs from each participant. Both experts reviewed synchronized multimodal PSG signals and were blinded to the TAS signals, model outputs, automatically detected RIP troughs, and each other’s annotations. Annotation discrepancies were jointly reviewed and resolved by consensus. Detailed sampling, annotation, blinding, adjudication, and agreement procedures are provided in Supplementary Note S1.

The final expert reference contained 1428 respiratory-onset landmarks. The median absolute difference from the frozen automatic RIP troughs was 0.125 s (IQR, 0.062–0.250 s), with 76.8% and 94.0% of expert annotations falling within ±0.25 and ±0.50 s of a trough, respectively. The automatic trough labels achieved event-level F1 scores of 0.873 and 0.896 at matching tolerances of ±0.50 and ±1.00 s, respectively. In addition, 96.4% of expert annotations were temporally closer to a trough than to a peak of the processed RIP signal.

#### 2.3.3. Automated Landmark Extraction and Gaussian Weak Labels

**Figure 2:**
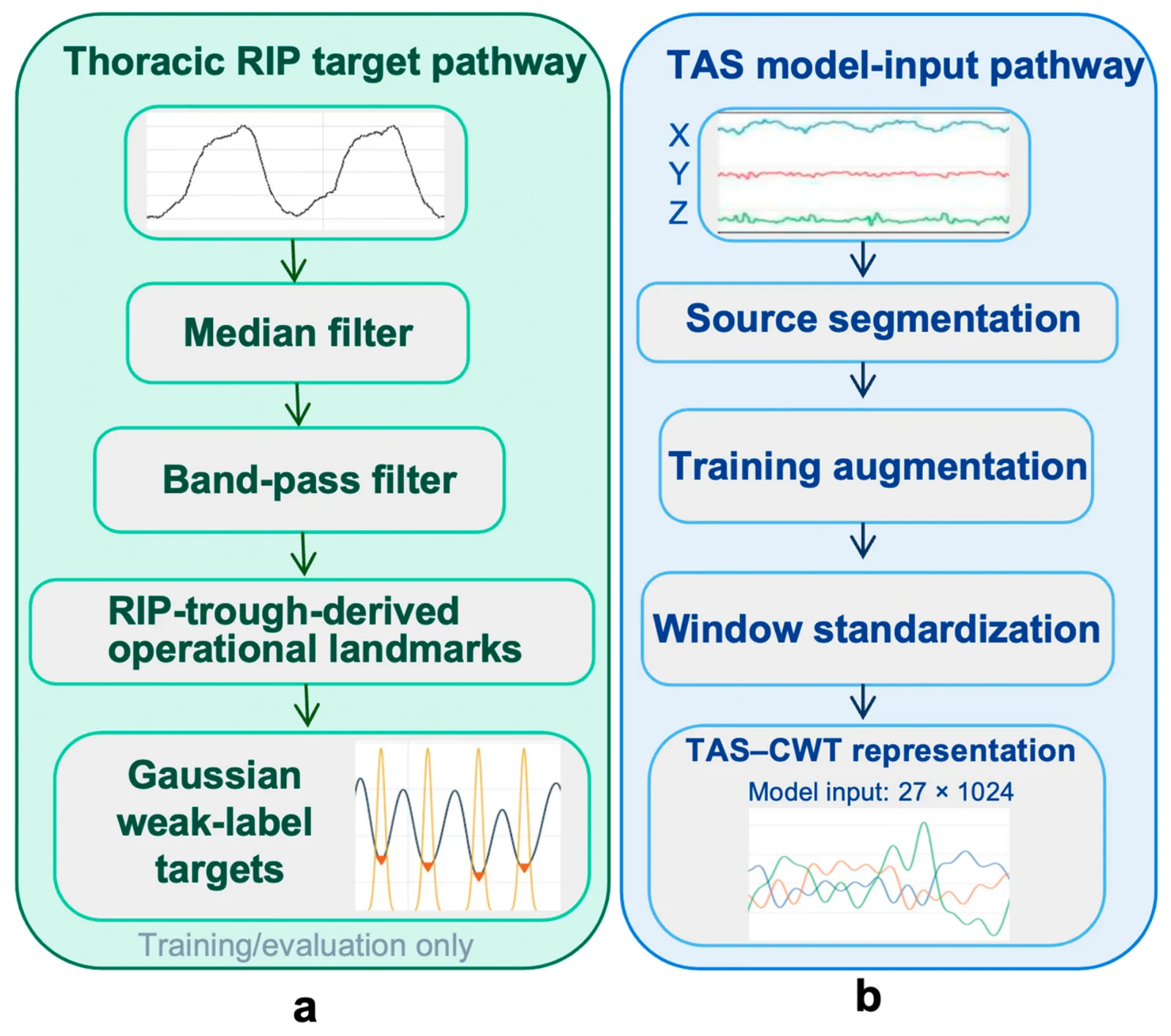
Construction of RIP-derived weak-label targets and TAS model inputs. (**a**) Thoracic RIP signals were filtered to extract trough-derived operational phase landmarks and generate Gaussian weak-label targets. (**b**) Triaxial accelerometry signals were segmented, standardized, and transformed into 27 × 1024 TAS–CWT representations. Data augmentation was applied during training only. RIP-derived targets were used for model training and post-inference scoring but were not provided as model inputs. CWT, continuous wavelet transform; RIP, respiratory inductance plethysmography; TAS, triaxial accelerometry sensor.

Before PSG recording, biocalibration and polarity/phase verification were performed for both thoracic and abdominal RIP channels, and recording-level signal quality was checked for each overnight study; the calibrated thoracic RIP trough was subsequently used as an operational respiratory-phase landmark representing the transition from end expiration to subsequent inspiration [39]. The landmark was used as the target for weak-label generation rather than as a manually adjudicated physiological reference standard (Figure 2a).

Candidate respiratory landmarks were identified as local troughs in the preprocessed thoracic RIP signal. Trough detection was performed using up to 10 s of additional context on both sides of each segment, with the context truncated at the recording boundaries. Candidates were required to have amplitudes no greater than the median minus 0.8 times the median absolute deviation of the standardized RIP signal. A minimum inter-trough interval of 1.5 s (48 samples at 32 Hz) and a minimum prominence of 0.1 were applied. Only troughs located within the central segment were retained to avoid duplicate assignment between adjacent segments.

Each retained trough was treated as an automatically extracted RIP-trough-derived operational phase landmark. To account for uncertainty in its temporal location, a unit-amplitude Gaussian weak label was centered at each landmark [40]:

(1) $$y\left(t\right)=\underset{k}{\max}\;k\left\{exp\left[-\frac{{\left(t-t_{k}\right)}^{2}}{2\sigma^{2}}\right]\right\},\;\sigma =0.2\;s\;\;\;$$

where $t_{k}$ denotes the location of the k thoracic RIP trough. Each Gaussian was truncated at ±4σ, corresponding to 26 samples on either side at 32 Hz. Overlapping Gaussian targets were combined using their pointwise maximum, resulting in a continuous training target ranging from 0 to 1.

#### 2.3.4. TAS Signals Preprocessing and Feature Representation

The TAS signals were analyzed at 32 Hz. For subsequent component-model training and standard-grid inference, each continuous sequence was partitioned into 32-s windows (1024 samples) with a 16-s stride (512 samples), resulting in 50% overlap between adjacent windows. The X-, Y-, and *Z*-axis signals were standardized independently within each window using z-score normalization, with the standard deviation bounded below at 0.01 for numerical stability. Continuous wavelet transform (CWT) coefficients were then computed from each standardized axis using the Mexican-hat wavelet at scales 8, 12, 16, 24, 32, 48, 64, and 80. Each scale-specific CWT channel was standardized within the same window and concatenated with the three time-domain channels, yielding a 27 × 1024 input representation.

Retained terminal sequences shorter than 1024 samples were reflect-padded before feature construction, and padded positions were excluded from loss calculation and performance evaluation using a valid-sample mask. Only the resulting TAS-derived representation was supplied to the component neural networks (Figure 2b).

### 2.4. Deep Learning Model Development

#### 2.4.1. Component Conformer Architectures

**Figure 3:**
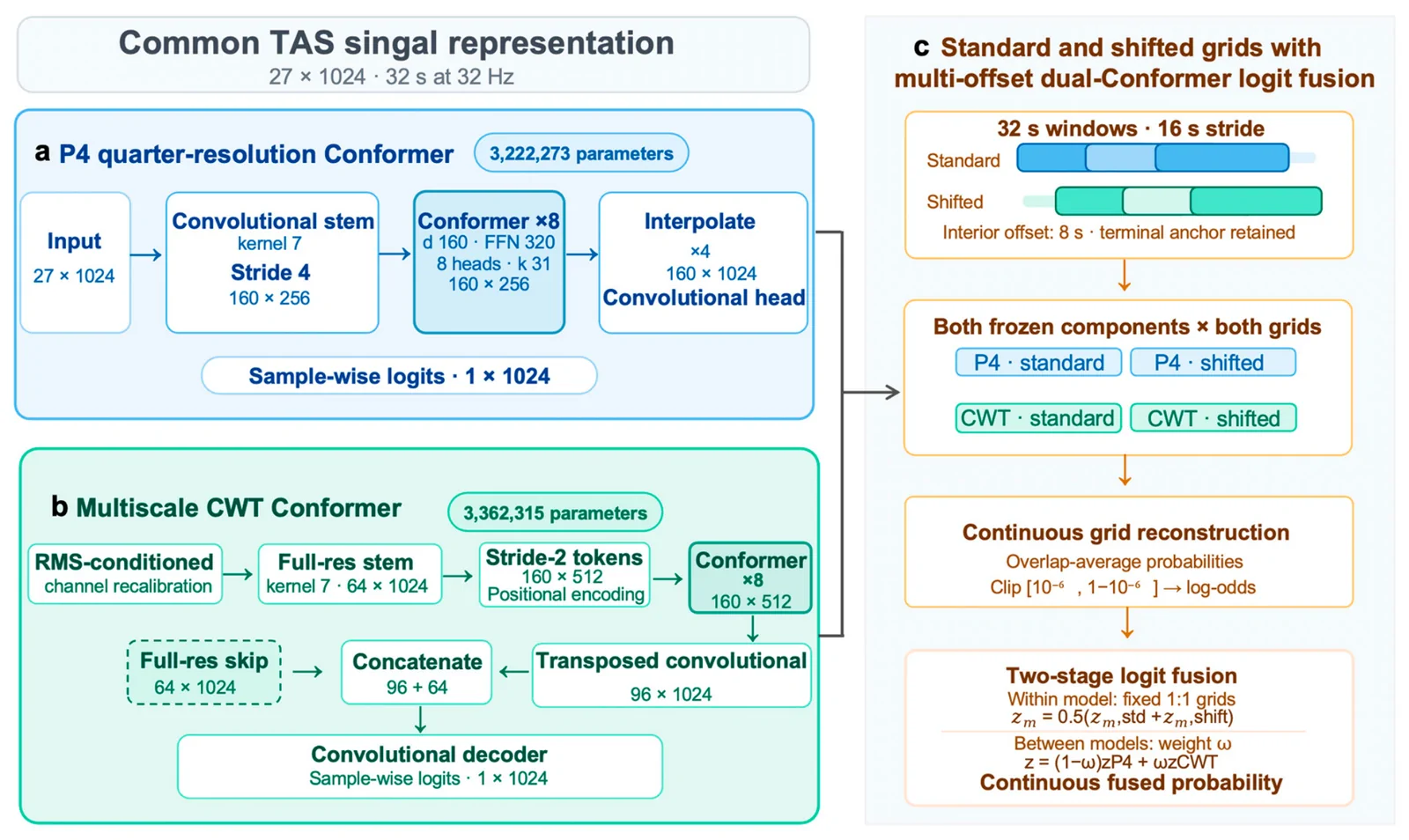
Component Conformer architectures and multi-offset logit fusion. (**a**) The P4 quarter-resolution Conformer generated sample-wise logits through stride-4 encoding and fourfold interpolation. (**b**) The Multiscale CWT Conformer combined stride-2 Conformer features with a full-resolution skip pathway. (**c**) Both frozen component models were applied to standard and 8-s-shifted window grids. Overlap-averaged probabilities were converted to logits, averaged with equal weights across grids within each model, and combined using the validation-selected model weight ω. CWT, continuous wavelet transform; TAS, triaxial accelerometry sensor.

Two component Conformer networks transformed the 27 × 1024 TAS signal representation into one logit per 32-Hz sample. The first component, the P4 quarter-resolution Conformer, contained 3,222,273 trainable parameters. A kernel-7, stride-4 convolutional stem projected the input to 160 channels at 256 temporal positions, followed by eight Conformer blocks with a model dimension of 160, feed-forward dimension of 320, eight attention heads, and a depthwise convolution kernel of 31. The resulting features were linearly interpolated to 1024 positions and converted to a single-channel logit sequence through a convolutional prediction head (Figure 3a).

The second component, the Multiscale CWT Conformer, contained 3,362,315 trainable parameters and retained a higher temporal resolution. An RMS-conditioned channel-recalibration module was first applied to the standardized input, followed by a kernel-7 convolutional stem that generated 64 full-resolution feature channels. These features formed both a full-resolution skip pathway and a stride-2 token pathway with 160 channels at 512 temporal positions. After sinusoidal positional encoding and eight Conformer blocks, the token representation was restored to 1024 positions by transposed convolution and concatenated with the full-resolution features. A convolutional decoder then produced one logit per sample (Figure 3b). Both component networks were optimized against the same thoracic RIP-trough-derived weak-label target.

#### 2.4.2. Multi-Offset Dual-Conformer Logit Fusion

For each retained sequence, two deterministic inference grids were constructed from the 32 s windows defined previously. The standard grid began at sample 0 and advanced by 512 samples (16 s), with an additional window anchored at the final valid start when required. The shifted grid retained the start and terminal anchors to ensure complete coverage, while its interior windows began at sample 256 (8 s) and advanced by 512 samples. Each component model was applied separately to both grids (Figure 3c). Within each model–grid combination, probabilities from overlapping windows were averaged sample by sample to reconstruct a continuous probability sequence covering all valid samples. Only the temporal placement of the windows differed between the two views; feature construction and component-model parameters remained unchanged.

The reconstructed probabilities were clipped to ([10^−6^,1–10^−6^]) before conversion to log-odds. Let S and H denote the standard and shifted grids, respectively. The log-odds transformation was defined as follows [41]:

(2) $$let\;l\left(p\right)=\ln\left[\frac{p}{\left(1-p\right)}\right]\;\;\;$$

The standard and shifted grids were assigned fixed equal weights within each component model, after which the P4 and Multiscale CWT Conformer representations were combined using the between-model weight ω. The following fusion rule was specifically defined for the present study:

(3) $$z_{fusion}(t)=\frac{1-\omega }{2}\left\{l\left[p_{P4,S}(t)\right]+l\left[p_{P4,H}(t)\right]\right\}+\frac{\omega }{2}\left\{l\left[p_{CWT,S}(t)\right]+l\left[p_{CWT,H}(t)\right]\right\}$$

The continuous fused probability was then obtained using the standard inverse-logit, or logistic sigmoid, transformation [41]:

(4) $$p_{fusion}\left(t\right)=sigmoid\left[z_{fusion}\left(t\right)\right]$$

Thus, within-model grid fusion was performed using fixed 1:1 weighting in log-odds space, whereas ω controlled the relative contributions of the two component models.

#### 2.4.3. Component-Model Training and Checkpoint Freezing

Both component models were trained separately within each outer fold. Training windows were sampled using a participant-balanced strategy with a batch size of 64. The objective combined binary cross-entropy and soft Dice losses with equal weights, and the valid-sample mask was applied so that padded terminal samples did not contribute to the loss. AdamW optimization was used with a weight decay of 1 × 10^−4^ and gradient-norm clipping at 5. During component-model training only, the raw TAS signal windows were augmented by uniform gain scaling (0.8–1.2; probability 0.5), Gaussian noise scaled to the within-window channel standard deviation (relative scale 0.01; probability 0.5), random three-dimensional rotation (probability 0.8), conditional reflection within the rotation transform (probability 0.5), and axis-sign inversion (probability 0.2). All augmentations were disabled during validation and subsequent fusion inference.

The parameter-matched Conformer was trained with an initial learning rate of 1 × 10^−3^. Its learning rate was reduced by a factor of 0.5 after five validation epochs without improvement, with a maximum of 100 epochs and an early-stopping patience of 10 epochs. The Multiscale CWT Conformer used an initial learning rate of 3 × 10^−4^, a five-epoch warm-up followed by cosine decay, a maximum of 80 epochs, and an early-stopping patience of 12 epochs. Each component configuration was trained once using a fixed random seed of 2026. For each component and outer fold, the checkpoint yielding the highest event-level F1 on the designated inner-validation participant at a fixed threshold of 0.50 was retained. During multi-offset fusion inference, the corresponding checkpoint was loaded with strict state-dictionary matching and used in evaluation mode without gradient computation. The checkpoint parameters were not updated, and neither joint fine-tuning nor an additional trainable fusion layer was introduced.

### 2.5. Experimental Design and Model Validation

#### 2.5.1. Subject-Level Outer Leave-One-Subject-Out Validation

**Figure 4:**
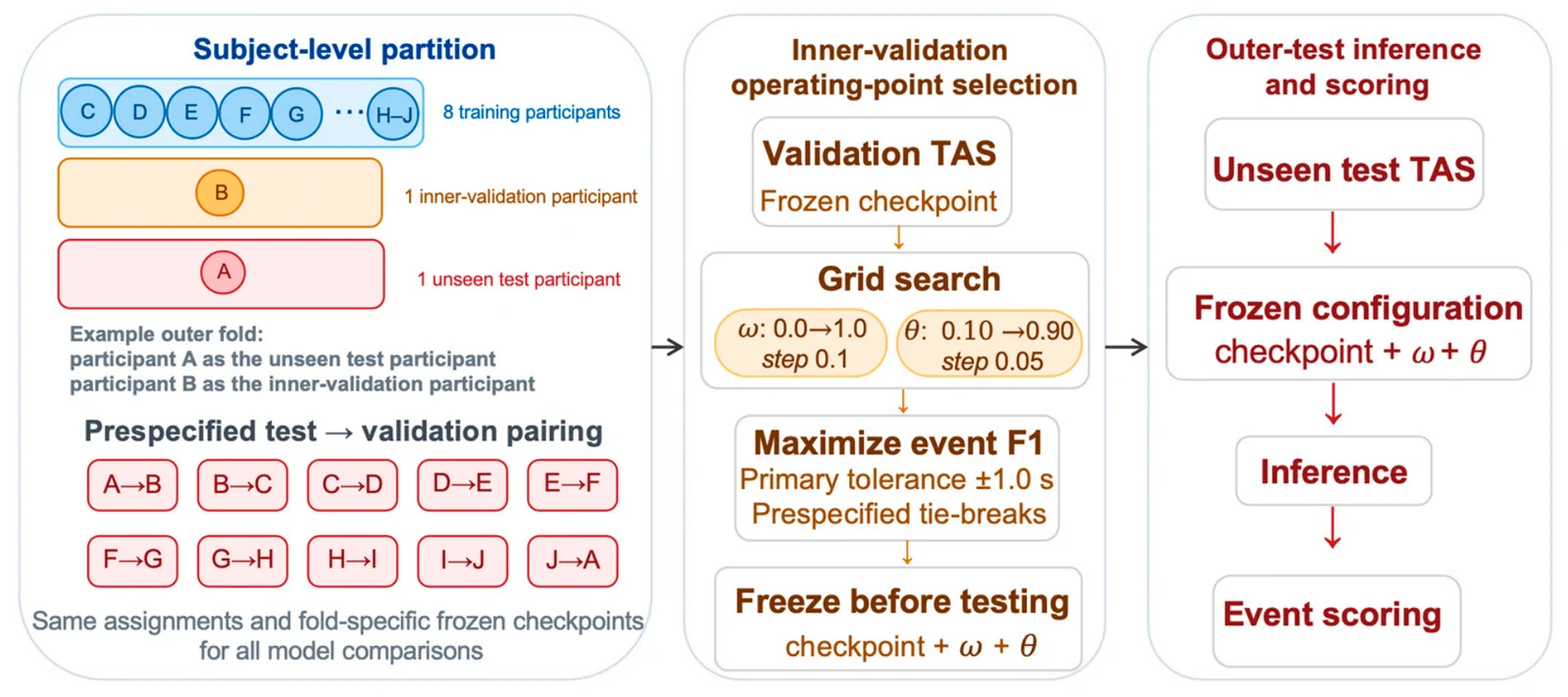
Subject-level outer-LOSO validation and operating-point selection. In each outer fold, eight participants were used for training, one prespecified participant was used for inner validation, and one unseen participant was used for outer testing. Using the frozen component checkpoints, the model weight ω and event threshold θ were selected on the inner-validation participant by maximizing event-level F1 at the primary ±1.0 s tolerance. The checkpoints and operating point were then frozen before outer-test inference. Outer-test RIP-derived landmarks were used only for post-inference scoring. LOSO, leave-one-subject-out; RIP, respiratory inductance plethysmography; TAS, triaxial accelerometry sensor.

Outer leave-one-subject-out (LOSO) validation was used to evaluate generalization to unseen participants (Figure 4). In each of the ten outer folds, one participant was held out for testing, one of the remaining nine was designated for inner validation, and the other eight formed the training set. For outer-test participants A–J, the corresponding inner-validation participants were B, C, D, E, F, G, H, I, J, and A, respectively. Participant assignment was completed before segmentation and overlapping-window generation; therefore, all recording segments and derived windows from a given participant remained within the same subset. Participant identifiers were used only for data partitioning and were not included among the model inputs. The same participant assignments and corresponding fold-specific frozen checkpoints were retained across the single-component, single-grid fusion, and multi-offset fusion comparisons. Inner selection was thus based on one prespecified validation participant per outer fold rather than a complete inner subject-level cross-validation.

Validation and test inference used only TAS-derived model inputs. For each outer fold, the Multiscale CWT Conformer logit weight was evaluated from 0.0 to 1.0 in increments of 0.1, together with event thresholds ranging from 0.10 to 0.90 in increments of 0.05, exclusively on the prespecified inner-validation participant. The candidate pair yielding the highest event-level F1 under the primary ±1.0 s matching tolerance was retained. Ties were resolved sequentially by higher recall, higher precision, a model weight closer to 0.5, and a threshold closer to 0.5. The selected weight and threshold were recorded and frozen before outer-test inference. No information from the outer-test weak labels contributed to checkpoint retention or operating-point selection; these labels were used only for post-inference scoring.

#### 2.5.2. Comparator Models and Fair-Comparison Controls

The proposed multi-offset dual-conformer fusion was evaluated against three groups of comparators. First, the parameter-matched Conformer and the Multiscale CWT Conformer were evaluated separately on the standard grid. Second, a single-grid dual-conformer fusion used the same frozen component checkpoints, cross-model logit-fusion rule, candidate weight and threshold grids, post-processing procedure, and subject-level validation folds as the proposed method, but omitted the shifted grid. This comparison was used to estimate the incremental contribution of multi-offset inference while keeping the component models and fusion procedure unchanged. Third, the single-model comparison set comprised the parameter-matched Conformer and seven additional approximately parameter-matched baselines: U-Net, dilated TCN, CNN–BiLSTM, time-series Transformer, ResNet-1D, dilated Conv–GRU, and Fourier mixer. All single models received the same 27-channel TAS–CWT representation.

Model sizes and fusion parameterization are summarized in Supplementary Table S1. All comparators used the same weak-label targets and followed the common subject-level partitioning and event-evaluation protocols. Because the dual-model configurations had greater total capacity and retained model-specific training schedules, the comparisons reflect the complete implemented configurations rather than isolated architectural effects.

#### 2.5.3. Ablation Analysis of the Multiscale CWT Conformer

Four ablation variants of the Multiscale CWT Conformer were retrained across all outer folds, each using the same fixed random seed. The variants comprised: (1) joint removal of channel recalibration and positional encoding; (2) removal of the high-resolution branch; (3) removal of all CWT-derived channels, leaving only the three standardized time-domain TAS channels; and (4) replacement of the Gaussian weak labels with single-sample hard targets at the reference thoracic RIP troughs. Except for the designated modification, the subject-level partitions, data augmentation, optimization, checkpoint-selection, and event-evaluation procedures were kept consistent with those of the complete component model. For the hard-target variant, the same equally weighted binary-cross-entropy and soft Dice objective was retained so that the comparison differed in target representation rather than in the loss function. Each ablation was evaluated at the component-model level and was not incorporated into the multi-offset fusion.

#### 2.5.4. Descriptive Analyses of the Multiscale CWT Component by Sleep Stage and Body Position

Exploratory descriptive analyses were performed for the Multiscale CWT Conformer component only. Epoch-wise sleep-stage and body-position annotations at 30 s resolution were temporally aligned with the complete-record standard-grid predictions for each outer-test participant. The annotations were used only for post-inference stratification and were not provided as model inputs or used for training, checkpoint retention, or operating-point selection. These analyses were not repeated for the multi-offset fusion.

Sleep stages were categorized as N1, N2, N3, rapid eye movement (REM) sleep, and body positions as right lateral, supine, left lateral. For each stratum, the reported number of participants represented outer-test participants contributing valid annotated data rather than the full cohort. Strata with limited annotated duration or few reference landmarks were reported descriptively and were not used for model selection or formal statistical inference.

#### 2.5.5. Post-Hoc Illustrative Interval Analysis of the Multiscale CWT Component

After component-model inference, operating-point selection, and event matching had been completed, four 60 s intervals were selected post hoc from the standard-grid outer-test outputs of the Multiscale CWT Conformer. The intervals were chosen to illustrate concordant detection, false-positive predominance, false-negative predominance, and disagreement between model detections and RIP-trough-derived operational phase landmarks during irregular thoracic RIP morphology. Selection was based on interval-level event-error counts and documented signal characteristics and was performed solely for qualitative visualization.

#### 2.5.6. Sampled Streaming Replay of the Multiscale CWT Component

A sampled streaming replay was conducted for the Multiscale CWT Conformer component only. For each outer-test participant, three 5 min blocks were extracted at approximately 20%, 50%, and 80% of the recording duration. A trailing 32 s TAS window was updated at 1 s intervals, and only the predictions corresponding to the newly available final second were passed to the event-detection logic. In the mode without additional look-ahead beyond the window-update time, the probabilities were processed using a causal exponential moving average with a time constant of 0.1 s, followed by a 0.25 s confirmation interval and a 1.0 s refractory interval. A fold-specific event threshold was selected from two 5 min blocks of the corresponding inner-validation participant and frozen before replay of the outer-test blocks. A separate exploratory condition allowed 1 s of additional look-ahead. The multi-offset fusion was not evaluated in this analysis.

No raw TAS sample acquired after a given window-update time was included in the corresponding model input. CWT features were recomputed independently within each finite trailing window without external signal padding, and event decisions were based on predictions at the right edge of the window. The causal exponential moving average used in the replay replaced the symmetric Gaussian probability smoothing applied in the offline complete-record analysis. Thus, the replay preserved the information boundary at each update time but did not reproduce the feature-boundary and post-processing conditions of the offline analysis.

Confirmed detections were matched one-to-one to reference thoracic RIP troughs within a tolerance of ±1.0 s. Confirmation delay was defined as the interval from the matched reference trough to the confirmed model output. Computational performance was summarized by the ratio of processing time to replayed duration, together with single-window latency measured in 20 windows per outer fold. This retrospective GPU-based analysis of sampled recording blocks was intended as an algorithmic feasibility assessment rather than an end-to-end evaluation of prospective real-time deployment.

### 2.6. Event Post-Processing, Performance Metrics, and Statistical Analysis

For complete-record evaluation, the fused probability sequence was smoothed using a symmetric one-dimensional Gaussian filter with a standard deviation of two samples. Predicted events were identified as local probability peaks meeting the fold-specific threshold selected on the inner-validation participant, with a minimum inter-event interval of 1.0 s. Reference events were recovered as local maxima of the precomputed Gaussian weak-label sequence at a height of at least 0.50; these maxima corresponded to the reference thoracic RIP troughs. Predictions and reference events were matched chronologically on a one-to-one basis within a tolerance of ±1.0 s. Event-level precision, recall, F1, and mean absolute timing error for matched events were calculated, with F1 at the ±1.0 s tolerance designated as the primary algorithmic endpoint.

Sensitivity analysis repeated event matching using a tolerance of ±0.5 s. Unmatched predictions per analyzed minute and unmatched reference troughs per 100 reference events were additionally reported as descriptive measures of error burden. Sample-wise accuracy was retained as a supplementary metric because of its sensitivity to class imbalance. Fold-specific values and participant-level mean ± standard deviation were reported. Micro-F1 was calculated by pooling event counts across outer-test participants and was treated as a descriptive summary; the participant remained the independent unit for paired statistical inference.

The participant-mean F1 score of the proposed method and the paired participant-level F1 differences between the proposed method and the single-grid dual-model fusion and the two component models were summarized using 20,000-replicate participant-bootstrap 95% confidence intervals. Paired differences were evaluated using exact two-sided sign-flip tests, with Holm adjustment across the three prespecified comparisons. The Multiscale CWT Conformer ablations were analyzed using paired Wilcoxon signed-rank tests with Holm adjustment. Sleep-stage, body-position, illustrative-interval, and sampled streaming-replay analyses were descriptive and were not subjected to confirmatory statistical testing.

## 3. Results

### 3.1. Study Population and Analyzable Data

Ten participants, comprising eight men and two women, were included, and all participants were of Han Chinese ethnicity. Their mean age was 36.9 ± 11.6 years, with a range of 23–56 years. The demographic and clinical characteristics of the individual participants are summarized in Table 1. Complete synchronized recordings comprised 5377.3 min and 1078 consecutive source segments. The outer LOSO evaluation included 19,359 standard-grid TAS windows and 75,499 automatically generated thoracic RIP-trough landmarks. All eligible synchronized samples were retained, and reconstructed test coverage was 100% in every outer fold (Table 2). The independent units for generalization inference were the 10 participants rather than the individual landmark events.

### 3.2. Primary Outer-LOSO Performance and Event-Matching Sensitivity

**Figure 5:**
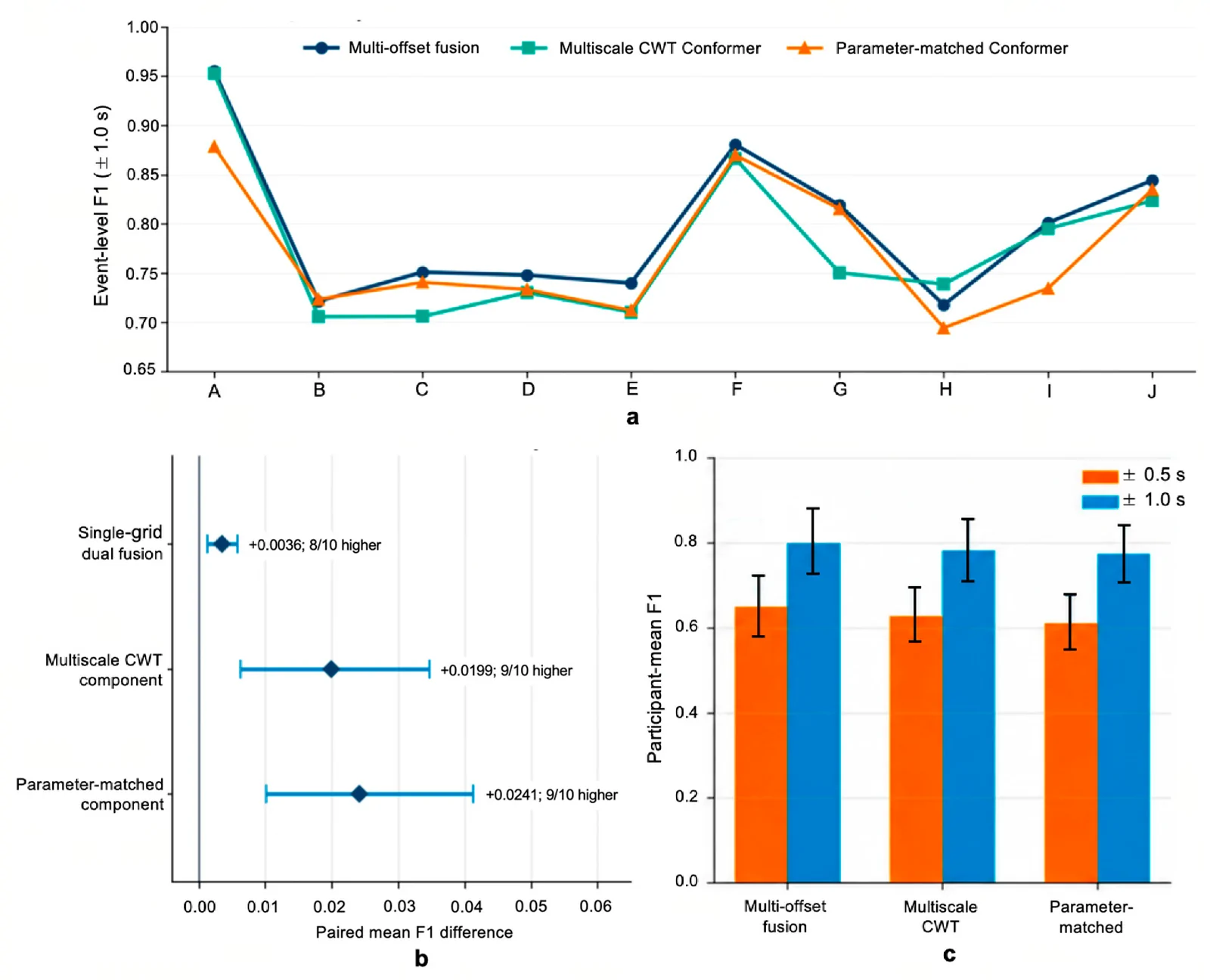
Outer-LOSO performance of the multi-offset dual-conformer fusion. (**a**) Participant-level event F1 scores at the ±1.0 s matching tolerance for the proposed fusion and its two component models. (**b**) Paired participant-mean F1 differences between the proposed fusion and each comparator. Points denote mean differences, and error bars denote participant-bootstrap 95% confidence intervals; positive values favor the proposed fusion. (**c**) Participant-mean F1 scores at the ±0.5 and ±1.0 s matching tolerances. Bars and error bars represent mean ± standard deviation across the ten held-out participants. CWT, continuous wavelet transform; LOSO, leave-one-subject-out.

At the primary ±1.0 s matching tolerance, the multi-offset dual-conformer fusion achieved a participant-mean precision of 0.78 ± 0.09, recall of 0.82 ± 0.08, F1 score of 0.80 ± 0.08 (bootstrap 95% CI, 0.75–0.85), accuracy of 0.82 ± 0.04, and matched-event timing mean absolute error of 0.300 s. Pooled event counts comprised 61,738 true positives, 17,858 false positives, and 13,761 false negatives, corresponding to a micro-F1 of 0.80 (Figure 5).

When the matching tolerance was reduced to ±0.5 s, the participant-mean F1 score decreased to 0.65 (Figure 5c). At ±1.0 s, participant-level F1 scores ranged from 0.72 to 0.96. The validation-selected Multiscale CWT logit weights ranged from 0.20 to 0.70, and the corresponding event thresholds ranged from 0.15 to 0.30; fold-level selections and outer-test results are provided in Supplementary Table S2.

### 3.3. Comparative Performance of Complete Model Configurations and Inference Ensembles

Among the complete-record configurations, the multi-offset dual-conformer fusion had the highest observed participant-mean F1 score. The remaining parameter-matched single-model baselines yielded mean F1 scores ranging from 0.72 to 0.76 (Table 3). Compared with the Multiscale CWT Conformer, the multi-offset fusion increased mean F1 by 0.0199 (bootstrap 95% confidence interval [CI], 0.0063–0.0347) and achieved a higher F1 score in 9 of 10 participants (exact sign-flip *p* = 0.0254; Holm-adjusted *p* = 0.0352). Compared with the parameter-matched Conformer, the corresponding mean F1 difference was 0.0241 (95% CI, 0.0101–0.0413), again with higher F1 in 9 of 10 participants (exact sign-flip *p* = 0.0039; Holm-adjusted *p* = 0.0117). Relative to the otherwise identical single-grid dual-conformer fusion, inclusion of the shifted grid increased mean F1 by 0.0036 (95% CI, 0.0013–0.0058), with improvement in 8 of 10 participants (exact sign-flip *p* = 0.0176; Holm-adjusted *p* = 0.0352). This observed increase was smaller than the prespecified exploratory advancement margin of 0.005.

### 3.4. Ablation Results of the Multiscale CWT Conformer

Ablation results for the Multiscale CWT component are summarized in Table 4. Removing individual architectural components or all CWT-derived channels resulted in relatively small reductions in participant-mean F1, with none of the corresponding confidence intervals excluding zero. In contrast, replacing the Gaussian weak labels with single-sample hard labels produced the largest deterioration, mainly through reduced recall, and was the only ablation that remained significant after Holm correction. These analyses were limited to the Multiscale CWT component and did not evaluate the complete dual-model fusion.

### 3.5. Descriptive Performance Across Sleep Stages and Body Positions

The descriptive sleep-stage and body-position analyses included the standard-grid outer-test outputs of participants A, B, E, F, G, H, I, and J. Pooled F1 scores were 0.88 in N3, 0.83 in N2, 0.78 in rapid eye movement sleep, 0.72 in N1, and 0.68 during wakefulness (Supplementary Table S3). Participant-mean F1 was highest in N2 (0.84 ± 0.09), whereas the corresponding value in N3 was 0.82 ± 0.11. During scored sleep, pooled F1 was 0.84 in the supine position, 0.83 in the left-lateral position, and 0.78 in the right-lateral position. Left-lateral duration was contributed by three participants, but only two had reference landmarks. These subgroup results were descriptive and were obtained for the Multiscale CWT component only.

### 3.6. Post-Hoc Illustrative Interval Analysis

**Figure 6:**
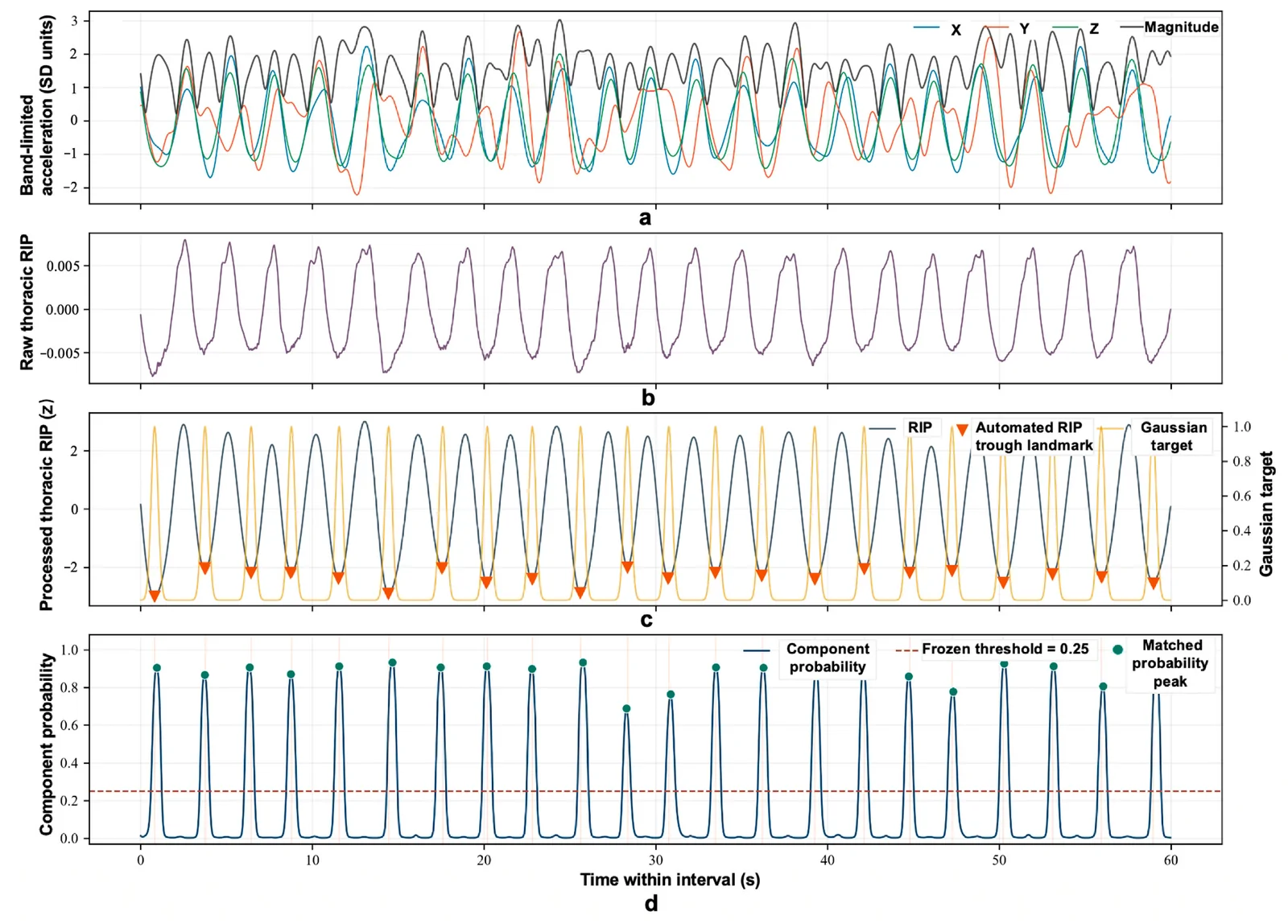
Representative high-agreement interval for the Multiscale CWT Conformer. A 60-s standard-grid outer-test interval from Participant I is shown. (**a**) Band-limited triaxial acceleration signals and vector magnitude. (**b**) Raw thoracic respiratory inductance plethysmography (RIP). (**c**) Processed thoracic RIP with RIP-trough-derived operational phase landmarks and Gaussian targets. (**d**) Component probability, the frozen fold-specific threshold, and matched probability peaks. All 22 landmarks were matched within the prespecified temporal tolerance (TP = 22, FP = 0, FN = 0; F1 = 1.00).

The post-hoc intervals illustrated four recurrent output patterns of the Multiscale CWT component: close alignment between TAS-derived probability peaks and RIP-trough-derived operational phase landmarks during regular respiratory modulation; additional probability peaks during transient multiaxial activity; missed landmarks when TAS respiratory modulation was weak; and disagreement during irregular RIP morphology (Figure 6 and Figures S1–S3).

In the high-agreement example shown in Figure 6, recurring respiratory modulation was visible in the band-limited triaxial acceleration signals, while the thoracic RIP signal exhibited a comparatively regular respiratory pattern. The component-probability peaks closely followed the RIP-trough-derived operational phase landmarks throughout the interval. This example illustrates the temporal correspondence achieved under regular respiratory morphology but, as a post-hoc selected interval, should not be interpreted as representative of complete-record performance.

In the false-negative-dominant example, weaker and less regular TAS respiratory modulation coincided with numerous RIP-derived landmarks that lacked corresponding model-probability peaks, together with several additional unmatched predictions (Supplementary Figure S1). In the reference-empty example, the raw thoracic RIP remained nearly constant and generated no RIP-trough-derived landmarks, whereas multiple model-probability peaks crossed the frozen threshold in the presence of transient multiaxial activity (Supplementary Figure S2). Because the reference sequence contained no landmarks, these detections should be interpreted as unmatched predictions relative to the RIP-derived reference rather than independently verified physiological false-positive events. In the final example, the thoracic RIP signal rapidly attenuated toward a near-constant baseline, producing only one operational phase landmark, while the model output contained multiple threshold-crossing peaks (Supplementary Figure S3). This interval illustrates how abnormal or attenuated reference-signal morphology can contribute to disagreement between TAS-derived model outputs and RIP-derived landmarks.

All four intervals were selected after completion of the quantitative evaluation solely for qualitative illustration and were not used for model selection or estimation of overall or subgroup performance.

### 3.7. Sampled Strict-Streaming Replay

In the separate component-only replay, the zero-additional-look-ahead mode yielded a participant-mean precision of 0.76, recall of 0.80, and F1 score of 0.77. Median confirmation delay was 0.686 s, and the 95th-percentile delay was 1.508 s. The minimum replay coverage was 89.7% because the initial 32 s context was unavailable at the beginning of each sampled block (Table S4).

The archived replay summary reported a mean real-time factor of 0.00398, a 95th-percentile single-window latency of 0.0281 s, and a maximum measured GPU memory use of 26.7 MB. With 1 s of additional look-ahead, mean F1 increased to 0.78 and median confirmation delay increased to 1.744 s. These measurements apply to the sampled Multiscale CWT component replay and not to the complete multi-offset dual-conformer fusion.

## 4. Discussion

This single-center pilot algorithm-development study evaluated whether an operational respiratory-phase landmark derived from the thoracic RIP trough could be recovered from TASs in a previously unseen participant. Under outer LOSO validation, the training-free multi-offset fusion of two complementary Conformer configurations achieved the highest observed complete-record agreement among the evaluated configurations. The fusion yielded a higher F1 score in 9 of 10 participants relative to each component.

Independent multimodal PSG review showed close temporal correspondence between the frozen automatic RIP-trough landmarks and expert-identified respiratory onsets, supporting their use as operational reference events. Nevertheless, this validation involved only 10 participants and 100 min of sampled recordings, and the RIP-derived landmarks should not be interpreted as an error-free physiological gold standard. The reference and sensing modalities considered here represent related but non-interchangeable physiological quantities. In standard sleep scoring, an oronasal thermal sensor primarily indicates the presence or near absence of oral and nasal airflow for apnea detection, whereas nasal pressure provides a nonlinear surrogate of nasal airflow for hypopnea detection; thoracic and abdominal RIP describe respiratory excursions and their phase relationship, and calibrated or summed RIP may provide a volume-related surrogate rather than a direct airflow measurement [39]. In the same PSG recordings, Sabil et al. found that nasal pressure detected approximately 30% more apneas and RIPsum approximately 30% fewer apneas than an oronasal thermal sensor, with sensor-dependent differences in event duration and characteristic errors during oral breathing and thoracoabdominal paradox [42]. A TAS measures local surface acceleration caused by respiratory and non-respiratory chest-wall motion and is therefore still further removed from airflow than nasal pressure or a thermal sensor. Its advantages are low burden and sensitivity to multiaxial mechanical motion, whereas its limitations include posture dependence, attachment and orientation effects, motion contamination, and the absence of a unique one-to-one mapping from local acceleration to airflow or composite respiratory effort.

Previous accelerometry studies support the feasibility of deriving respiratory information from body-surface motion, but they have addressed different targets. A dual-triaxial-accelerometer system reconstructed thoracic and abdominal respiratory signals during sleep and was evaluated by 60 s epoch correlations with nasal airflow rather than by landmark timing [31]. Doheny et al. estimated respiratory rate in 30 s epochs from chest and upper-abdominal accelerometers in 11 participants, using PSG cannula flow as the benchmark [34]. Ryser et al. evaluated a single chest-worn accelerometer over more than 98 h in 13 healthy volunteers, reporting respiratory-rate error and classification of respiratory cessations rather than individual phase landmarks [18]. More recently, a deep-learning model trained on chest accelerometry in 146 participants estimated a continuous respiratory-effort waveform and detected breaths in a 72-participant holdout set [19]. Related phase-oriented work has localized and classified inspiration and expiration from suprasternal tracheal sounds and movements [17], while BreathFinder segmented complete respiratory-cycle intervals from thoracic RIP and reported an F1 score of approximately 0.94 using an interval-overlap criterion of greater than 80% on selected hand-annotated recordings [43]. These values should not be compared directly with the present F1 score. The studies differ in sensor location and number, target quantity, reference signal, manual versus automated labeling, point-event versus interval output, matching tolerance, selected segments versus complete nights, participant-level separation, and macro versus pooled aggregation. Accordingly, the present contribution is not a claim of numerical superiority over these methods; it is evidence that a single surface TAS input can recover a RIP-trough-derived operational phase landmark across complete recordings from same-center held-out participants.

Respiratory synchronization provides the clinical motivation for examining phase-related sensing in closed-loop HGNS. Respiration-synchronized unilateral systems use a thoracic sensing lead to coordinate stimulation with the respiratory cycle [3,4,5,6], and stimulation timing can influence how effectively tongue protrusion overlaps with inspiratory upper-airway vulnerability [44,45,46,47]. Avoiding stimulation outside the intended respiratory phase may also be relevant to patient comfort, muscle loading, and energy use. A surface TAS could reduce sensing burden during non-invasive development and may ultimately inform alternative respiratory sensing strategies. In the present framework, RIP was used only to generate training and evaluation landmarks, whereas inference in each held-out participant used the TAS alone. This separation supports investigation of TASs as an intended sensing modality, but it does not establish equivalence to an implanted thoracic sensing lead. The current study should therefore be interpreted as an upstream sensing study rather than a clinical-controller or stimulation-timing validation.

The use of cross-modal weak supervision enabled reference labels to be generated across 5377.3 min of complete synchronized recording, but the validity of the evidence chain still depends on the reference landmark. The acquisition system was calibrated before PSG to confirm the recorded polarity and phase relationships of the nasal-pressure, thermal-airflow, and thoracoabdominal RIP channels. This system-level calibration addresses the previous concern that an unverified polarity could reverse the interpretation of an RIP extremum. It does not, however, constitute event-by-event physiological adjudication.

The two component models showed performance profiles that were consistent with the rationale for output-level fusion. The Multiscale CWT Conformer had higher mean precision, whereas the parameter-matched Conformer had higher recall, and their validation-selected combination improved both measures relative to at least one component. The higher-resolution branch and multiscale CWT channels may help preserve local respiratory morphology and timing, whereas the more compressed Conformer representation may provide a complementary contextual decision boundary. Consistently, removing all CWT-derived channels reduced the Multiscale CWT component mean F1 from 0.78 to 0.75, but the paired confidence interval included zero. In contrast, replacing the Gaussian weak label with a single-sample hard target reduced recall from 0.78 to 0.52 and F1 from 0.78 to 0.63 indicating that a temporally distributed target was important for this single-seed component configuration. The shifted grid produced only a further 0.0036 increase in mean F1 over otherwise identical single-grid dual-conformer fusion, although the direction was positive in 8 of 10 participants. This small gain is compatible with reduced sensitivity to window placement, but it was below the prespecified exploratory advancement margin of 0.005 and does not establish confirmed superiority. The approach required no retraining or learned fusion layer, but it retained 6.58 million component parameters and approximately doubled inference relative to single-grid dual-model fusion.

The tolerance analysis further shows why an event F1 score should not be equated with HGNS triggering suitability. When the matching tolerance was reduced from ±1.0 s to ±0.5 s, the participant-mean F1 score of the proposed fusion decreased from 0.80 to 0.65. At the primary tolerance, pooled counts still included 17,858 unmatched predictions and 13,761 unmatched references, corresponding to 3.32 unmatched predictions per minute and 18.2 unmatched references per 100 landmarks. These quantities are not clinical false-trigger and missed-stimulation rates because no stimulation was delivered and the reference was not a clinical stimulation window. They nevertheless reveal an operational error burden that is not conveyed by F1 alone. The matched-event timing mean absolute error of 0.300 s was calculated only for successfully matched events and should likewise not be interpreted as the delay distribution for all respiratory cycles. Future evaluation should use an independently adjudicated reference, normalize timing error by respiratory-cycle duration, predefine a clinically meaningful phase window, and report the consequences of early, late, additional, and missed detections separately.

The descriptive stage- and position-stratified analyses suggest that TAS-derived respiratory information was not uniform across behavioral states. For the Multiscale CWT component, pooled F1 was higher in N2 and N3 sleep than during N1 sleep, and participant-mean F1 was highest in N2. This pattern may reflect more regular respiratory modulation and less non-respiratory movement during stable non-rapid-eye-movement sleep, but arousal burden, movement, respiratory-event type, and signal quality were not jointly modeled. During scored sleep, pooled F1 was 0.84 in the supine position, 0.83 in the left-lateral position, and 0.78 in the right-lateral position. Given the imbalance across sleep-stage and body-position subgroups, these exploratory analyses can only generate hypotheses about state- and position-dependent robustness and cannot establish that performance is invariant across sleep stages or body positions.

Several limitations constrain generalization and statistical interpretation. First, although the thoracic RIP trough was supported by expert review of the sampled epochs, it remained an operational proxy rather than a directly established physiological onset across the complete recordings. Second, the study included only 10 participants from a single center and one acquisition configuration, all of whom were of Han Chinese ethnicity, and the sex distribution was imbalanced, with eight male and two female participants. This small, sex-imbalanced, and ethnically homogeneous pilot cohort did not permit a reliable assessment of model performance across sex, ethnicity, or other demographic subgroups; therefore, subgroup-specific performance and broader population generalizability remain undetermined. The large number of landmarks increased event-level precision but did not replace the participant as the independent unit for generalization. Third, each component configuration was trained once with a single seed, so the fusion inherits unquantified training variability. Each outer fold used one rotating training-side participant for checkpoint selection and selection of the fusion weight and event threshold; this prevented direct use of the outer-test labels but did not provide full inner cross-validation.

Deployment evidence was also limited. The sampled replay evaluated only the Multiscale CWT component in three 5 min blocks per participant, not the multi-offset fusion. In the zero-additional-look-ahead mode, the component achieved a mean F1 score of 0.78, but confirmation delay and initial-context loss remained, and the archived GPU runtime and memory measurements cannot be extrapolated to a target CPU, digital signal processor, microcontroller, or implant. The complete fusion was not evaluated with strict causal preprocessing, quantified boundary effects, runtime and memory benchmarks, quantization, power measurement, stimulation artifacts, a control policy, or a safety analysis. Thus, surface sensing and faster-than-replay execution of one component should not be interpreted as implantable HGNS feasibility. Taken together, the findings support the pilot feasibility of recovering a calibrated thoracic RIP-trough-derived operational phase landmark from surface TAS in same-center unseen participants and suggest that complementary-model fusion can improve agreement without retraining. Independent multimodal label adjudication, locked external replication, multi-seed stability analysis, and causal device-oriented testing are required before the method can be interpreted as a respiratory controller input or a clinically actionable HGNS sensing strategy.

## 5. Conclusions

In this pilot study of 10 participants comprising 5377.3 min of synchronized overnight recordings, the proposed multi-offset dual-conformer fusion detected RIP-trough-derived operational phase landmarks from surface triaxial accelerometry with a participant-mean precision of 0.78 ± 0.09, recall of 0.82 ± 0.08, and F1 score of 0.80 ± 0.08 (bootstrap 95% CI, 0.75–0.85) at the prespecified ±1.0 s matching tolerance. The matched-event timing mean absolute error was 0.300 s. Moreover, reducing the matching tolerance to ±0.5 s decreased the participant-mean F1 score to 0.65. These results provide preliminary evidence that surface triaxial accelerometry can recover thoracic RIP-derived respiratory phase landmarks in unseen same-center participants, while also indicating that independent external validation and stricter timing evaluation are required before the method can be considered for respiratory-synchronized HGNS applications.

## Figures and Tables

**Table 1:** Demographic and clinical characteristics of the study population.

Subject	Sex	Age (y)	BMI (kg/m^2^)	SE (%)	Hypopneas (*n*)	AHI (Events/h)	ODI (Events/h)	OSA Severity
A	F	56	25.0	74.5	37	20.1	1.9	Moderate
B	M	52	26.9	60.5	40	24.0	11.7	Moderate
C	M	23	25.8	96.0	67	49.6	13.0	Severe
D	M	44	26.4	92.5	44	10.9	0.0	Mild
E	M	35	31.2	83.7	101	53.1	38.4	Severe
F	M	31	27.5	73.7	25	19.7	0.0	Moderate
G	M	23	31.7	92.4	177	51.2	54.5	Severe
H	M	27	26.6	94.5	30	48.5	17.1	Severe
I	F	35	23.9	85.6	4	1.3	0.0	Normal
J	M	43	26.6	90.7	209	60.8	42.5	Severe
Mean ± SD	—	36.9 ± 11.6	27.2 ± 2.5	84.4 ± 11.5	73.4 ± 68.5	33.9 ± 20.9	17.9 ± 20.1	——

SE, sleep efficiency; AHI, apnea–hypopnea index; BMI, body mass index; ODI, oxygen desaturation index; OSA, obstructive sleep apnea.

**Table 2:** Complete synchronized recordings and standard-grid windows analyzed in the outer LOSO evaluation.

Held-Out Subject	Duration (min)	300 s Segments	32 s Windows	Thoracic RIP Landmarks	Sample Coverage
A	398.9	80	1436	6159	100%
B	493.1	99	1775	7546	100%
C	462.9	93	1666	5852	100%
D	467.8	94	1684	5718	100%
E	568.8	114	2048	6398	100%
F	559.7	112	2015	8445	100%
G	639.0	128	2301	9396	100%
H	664.3	133	2392	8685	100%
I	609.4	122	2194	11,608	100%
J	513.4	103	1848	5692	100%
Total	5377.3	1078	19,359	75,499	100%

All eligible synchronized recordings were retained. Window counts describe the standard 16-s-stride grid used for training and conventional inference; the proposed method additionally used a deterministic 8-s-shifted interior grid with start and terminal boundary anchors. Landmark counts represent automatically extracted RIP-trough-derived operational phase landmarks. LOSO, leave-one-subject-out.

**Table 3:** Comparison of complete model configurations and inference ensembles.

Model	Parameters	Precision	Recall	F1 Score	Accuracy
Multi-offset dual-conformer fusion	6,584,588	0.78 ± 0.09	0.82 ± 0.08	0.80 ± 0.08	0.82 ± 0.04
Single-grid dual-conformer fusion	6,584,588	0.78 ± 0.09	0.81 ± 0.08	0.79 ± 0.08	0.83 ± 0.04
Multiscale CWT Conformer	3,362,315	0.78 ± 0.09	0.78 ± 0.10	0.78 ± 0.08	0.84 ± 0.04
Parameter-matched Conformer	3,222,273	0.75 ± 0.08	0.80 ± 0.08	0.77 ± 0.07	0.81 ± 0.03
Transformer	3,136,417	0.76 ± 0.08	0.77 ± 0.10	0.76 ± 0.08	0.82 ± 0.03
SE-U-Net	3,121,217	0.75 ± 0.08	0.78 ± 0.09	0.76 ± 0.08	0.81 ± 0.04
ResNet1D	3,248,201	0.74 ± 0.09	0.77 ± 0.08	0.76 ± 0.08	0.83 ± 0.04
Fourier Mixer	3,285,697	0.73 ± 0.07	0.77 ± 0.09	0.75 ± 0.06	0.81 ± 0.03
Dilated Conv-GRU	3,110,433	0.72 ± 0.06	0.76 ± 0.11	0.73 ± 0.07	0.80 ± 0.03
CNN-BiLSTM	3,103,745	0.70 ± 0.07	0.77 ± 0.10	0.73 ± 0.06	0.79 ± 0.05
TCN	3,043,665	0.71 ± 0.08	0.74 ± 0.07	0.72 ± 0.05	0.82 ± 0.04

Values are mean ± SD across ten held-out participants at validation-selected thresholds and ±1.0 s matching. Dual-model parameter counts are the sum of two frozen components; fusion added no trainable parameter. Inputs, weak labels, partitions, coverage, and scoring were shared, but component learning-rate schedules differed. CNN, convolutional neural network; GRU, gated recurrent unit; TCN, temporal convolutional network.

**Table 4:** Single-seed ablations of the Multiscale CWT component and weak-label configuration.

Condition	Parameters	Precision	Recall	F1 Score	Accuracy	F1 Difference [95% CI]
Multiscale CWT Conformer	3,362,315	0.78 ± 0.09	0.78 ± 0.10	0.78 ± 0.08	0.84 ± 0.04	—
Without channel attention and positional encoding	3,361,793	0.75 ± 0.08	0.79 ± 0.09	0.77 ± 0.08	0.82 ± 0.04	0.0058 [−0.0068, 0.0197]
Without high-resolution branch	3,222,795	0.75 ± 0.07	0.78 ± 0.09	0.76 ± 0.07	0.82 ± 0.04	0.0156 [−0.0096, 0.0449]
Without CWT features	3,351,100	0.74 ± 0.08	0.76 ± 0.07	0.75 ± 0.06	0.83 ± 0.03	0.0267 [−0.0105, 0.0841]
Hard point labels	3,362,315	0.81 ± 0.07	0.52 ± 0.10	0.63 ± 0.08	0.89 ± 0.02	0.1474 [0.1287, 0.1623] *

F1 difference is complete Multiscale CWT Conformer minus the ablation. * Holm-adjusted *p* = 0.0137; other adjusted comparisons were not significant. The hard-point condition used the same BCE-plus-Dice loss despite one-sample target sparsity. CWT, continuous wavelet transforms.

## Data Availability

The data supporting the findings of this study are available from the corresponding author upon reasonable request. The data are not publicly available due to privacy and ethical restrictions.

## Supplementary Materials

The following supporting information can be downloaded at https://www.mdpi.com/article/10.3390/bioengineering13101205/s1, Supplementary Note S1. Expert Validation of the RIP-Trough-Derived Operational Phase Landmarks. Supplementary Table S1. Model sources, architecture specifications, and output adaptations; Supplementary Table S2. Fold-level outer-test performance and training-side validation-only operating-point selection for multi-offset dual-conformer fusion; Supplementary Table S3. Descriptive landmark agreement across sleep stages and body positions during scored sleep for the Multiscale CWT component; Supplementary Table S4. Sampled strict-streaming replay of the Multiscale CWT Conformer component. Supplementary Figure S1. Representative false-negative-dominant interval for the Multiscale CWT Conformer. Supplementary Figure S2. Representative interval with unmatched predictions in the absence of RIP-derived landmarks. Supplementary Figure S3. Representative disagreement interval during irregular and attenuated thoracic RIP morphology. References [48,49,50,51,52,53,54] are cited in the supplementary materials.

## Abbreviations

The following abbreviations are used in this manuscript:

BiLSTM	Bidirectional long short-term memory
BMI	Body mass index
CI	Confidence interval
CNN	Convolutional neural network
CPU	Central processing unit
CWT	Continuous wavelet transform
DAC	Digital-to-analog converter
GPU	Graphics processing unit
GRU	Gated recurrent unit
HGNS	Hypoglossal nerve stimulation
LOSO	Leave-one-subject-out
LSB	Least significant bit
MEMS	Microelectromechanical systems
OSA	Obstructive sleep apnea
PAP	Positive airway pressure
PSG	Polysomnography
REM	Rapid eye movement
RIP	Respiratory inductance plethysmography
TAS	Triaxial accelerometry sensor
TASM	Triaxial acceleration sensor module

## References

[1] Cunningham J., Hunter M., Budgeon C., Murray K., Knuiman M., Hui J., Hillman D., Singh B., James A. (2021). The prevalence and comorbidities of obstructive sleep apnea in middle-aged men and women: The Busselton Healthy Ageing Study. J. Clin. Sleep Med..

[2] Schoch O.D., Baty F., Niedermann J., Rüdiger J.J., Brutsche M.H. (2014). Baseline predictors of adherence to positive airway pressure therapy for sleep apnea: A 10-year single-center observational cohort study. Respiration.

[3] Strollo P.J., Soose R.J., Maurer J.T., De Vries N., Cornelius J., Froymovich O., Hanson R.D., Padhya T.A., Steward D.L., Gillespie M.B. (2014). Upper-airway stimulation for obstructive sleep apnea. N. Engl. J. Med..

[4] Serghani M.M., Heiser C., Schwartz A.R., Amatoury J. (2024). Exploring hypoglossal nerve stimulation therapy for obstructive sleep apnea: A comprehensive review of clinical and physiological upper airway outcomes. Sleep Med. Rev..

[5] Hofauer B., Philip P., Wirth M., Knopf A., Heiser C. (2017). Effects of upper-airway stimulation on sleep architecture in patients with obstructive sleep apnea. Sleep Breath..

[6] Steffen A., Kilic A., König I.R., Suurna M.V., Hofauer B., Heiser C. (2018). Tongue motion variability with changes of upper airway stimulation electrode configuration and effects on treatment outcomes. Laryngoscope.

[7] Saltagi M.Z., Powell K., Saltagi A.K., Stahl S., Manchanda S., Parker N.P. (2023). Novel Outcome Analysis Tool for Hypoglossal Nerve Stimulator Sensor Lead Function and Comparison by Incision Type (2 Versus 3). Laryngoscope.

[8] Woodson B.T., Strohl K.P., Soose R.J., Gillespie M.B., Maurer J.T., de Vries N., Padhya T.A., Badr M.S., Lin H., Vanderveken O.M. (2018). Upper Airway Stimulation for Obstructive Sleep Apnea: 5-Year Outcomes. Otolaryngol. Head Neck Surg..

[9] Islam A.S., Pingree G., Chafin A., Fitzpatrick T.H., Nord R.S. (2024). Respiratory Sensing Lead Malfunction in Upper Airway Stimulation: A Single Institution Report. Laryngoscope.

[10] Alapati R., Wagoner S.F., Nieves A.B., Lawrence A., Rouse D., Larsen C. (2024). Upper airway stimulation device failure: A 7-year single center experience. Am. J. Otolaryngol..

[11] Bestourous D.E., Pasick L.J., Benito D.A., Zapanta P.E. (2020). Adverse events associated with the Inspire implantable hypoglossal nerve stimulator: A MAUDE database review. Am. J. Otolaryngol..

[12] Massaroni C., Nicolò A., Lo Presti D., Sacchetti M., Silvestri S., Schena E. (2019). Contact-Based Methods for Measuring Respiratory Rate. Sensors.

[13] Costanzo I., Sen D., Rhein L., Guler U. (2022). Respiratory Monitoring: Current State of the Art and Future Roads. IEEE Rev. Biomed. Eng..

[14] Uchiyama T., Nakayama S., Mohri K., Bushida K. (2009). Biomagnetic field detection using very high sensitivity magnetoimpedance sensors for medical applications. Phys. Stat. Sol. (A).

[15] Garcia M.A.C., Baffa O. (2015). Magnetic fields from skeletal muscles: A valuable physiological measurement?. Front. Physiol..

[16] Kozlov N.V., Volchkov S.O., Blyakhman F.A., Chestukhin V.V., Kurlyandskaya G.V. (2022). The Modeling of Magnetic Detection of Iron Oxide Nanoparticles in the Stream of Patient-Specific Artery With Stenotic Lesion: The Effects of Vessel Geometry and Particle Concentration. IEEE Trans. Magn..

[17] Montazeri Ghahjaverestan N., Kabir M., Saha S., Zhu K., Gavrilovic B., Alshaer H., Taati B., Yadollahi A. (2021). Automatic respiratory phase identification using tracheal sounds and movements during sleep. Ann. Biomed. Eng..

[18] Ryser F., Hanassab S., Lambercy O., Werth E., Gassert R. (2022). Respiratory analysis during sleep using a chest-worn accelerometer: A machine learning approach. Biomed. Signal Process. Control.

[19] Schipper F., van Sloun R.J.G., Grassi A., Overeem S., Fonseca P. (2023). A deep-learning approach to assess respiratory effort with a chest-worn accelerometer during sleep. Biomed. Signal Process. Control.

[20] Singh G.D., Battle J. (2025). Integrative treatment of obstructive sleep apnea: Principles and practice. J. Clin. Sleep Med..

[21] Guilleminault C., Stoohs R., Clerk A., Cetel M., Maistros P. (1993). A cause of excessive daytime sleepiness. The upper airway resistance syndrome. Chest.

[22] Akoumianaki E., Maggiore S.M., Valenza F., Bellani G., Jubran A., Loring S.H., Pelosi P., Talmor D., Grasso S., Chiumello D. (2014). The application of esophageal pressure measurement in patients with respiratory failure. Am. J. Respir. Crit. Care Med..

[23] Cantineau J.P., Escourrou P., Sartene R., Gaultier C., Goldman M. (1992). Accuracy of respiratory inductive plethysmography during wakefulness and sleep in patients with obstructive sleep apnea. Chest.

[24] Lloyd M., Reynolds D., Sheldon T., Stromberg K., Hudnall J.H., Demmer W.M., Omar R., Ritter P., Hummel J., Mont L. (2017). Rate adaptive pacing in an intracardiac pacemaker. Heart Rhythm.

[25] Schade C.M., Schultz D.M., Tamayo N., Iyer S., Panken E. (2011). Automatic adaptation of neurostimulation therapy in response to changes in patient position: Results of the Posture Responsive Spinal Cord Stimulation (PRS) Research Study. Pain Physician.

[26] Malekmohammadi M., Herron J., Velisar A., Bs Z.B., Trager M.H., Chizeck H.J., Brontë-Stewart H. (2016). Kinematic Adaptive Deep Brain Stimulation for Resting Tremor in Parkinson’s Disease. Mov. Disord..

[27] Liu G.Z., Guo Y.W., Zhu Q.S., Huang B.Y., Wang L. (2011). Estimation of respiration rate from three-dimensional acceleration data based on body sensor network. Telemed. J. E Health.

[28] Schipper F., van Sloun R.J.G., Grassi A., Derkx R., Overeem S., Fonseca P. (2021). Estimation of respiratory rate and effort from a chest-worn accelerometer using constrained and recursive principal component analysis. Physiol. Meas..

[29] Vennard H., Buchan E., Miller J., Kelly S., Cowan C., Meredith O., Henderson B., Lowe D.J., Patel N., Zuberi S. (2025). Respiratory monitoring and apnoea detection in paediatric and neonatal patients using a wearable accelerometer-based chest sensor: Protocol for an observational diagnostic feasibility study. BMJ Open.

[30] Nam Y., Kim Y., Lee J. (2016). Sleep Monitoring Based on a Tri-Axial Accelerometer and a Pressure Sensor. Sensors.

[31] Bricout A., Fontecave-Jallon J., Colas D., Gerard G., Pepin J.L., Gumery P.Y. Adaptive Accelerometry Derived Respiration: Comparison with Respiratory Inductance Plethysmography during Sleep. Proceedings of the 2019 41st Annual International Conference of the IEEE Engineering in Medicine and Biology Society (EMBC).

[32] Bricout A., Fontecave-Jallon J., Pépin J.L., Guméry P.Y. (2021). Accelerometry-derived respiratory index estimating apnea-hypopnea index for sleep apnea screening. Comput. Methods Programs Biomed..

[33] Morillo D., Rojas Ojeda J.L., Crespo Foix L.F., Jiménez A. (2010). An accelerometer-based device for sleep apnea screening. IEEE Trans. Inf. Technol. Biomed..

[34] Doheny E.P., Lowery M.M., Russell A., Ryan S. Estimation of respiration rate and sleeping position using a wearable accelerometer. Proceedings of the 2020 42nd Annual International Conference of the IEEE Engineering in Medicine & Biology Society (EMBC).

[35] Bates A., Ling M.J., Mann J., Arvind D.K. Respiratory Rate and Flow Waveform Estimation from Tri-axial Accelerometer Data. Proceedings of the 2010 International Conference on Body Sensor Networks.

[36] Liu S., Gao R.X., John D., Staudenmayer J., Freedson P. (2013). Tissue artifact removal from respiratory signals based on empirical mode decomposition. Ann. Biomed. Eng..

[37] Nassi T.E., Ganglberger W., Sun H., Bucklin A.A., Biswal S., van Putten M.J.A.M., Thomas R.J., Westover M.B. (2022). Automated Scoring of Respiratory Events in Sleep with a Single Effort Belt and Deep Neural Networks. IEEE Trans. Biomed. Eng..

[38] Romano C., Schena E., Formica D., Massaroni C. (2022). Comparison between Chest-Worn Accelerometer and Gyroscope Performance for Heart Rate and Respiratory Rate Monitoring. Biosensors.

[39] Berry R.B., Budhiraja R., Gottlieb D.J., Gozal D., Iber C., Kapur V.K., Marcus C.L., Mehra R., Parthasarathy S., Quan S.F. (2012). Rules for scoring respiratory events in sleep: Update of the 2007 AASM Manual for the Scoring of Sleep and Associated Events. J. Clin. Sleep Med..

[40] Newell A., Yang K., Deng J. (2016). Stacked Hourglass Networks for Human Pose Estimation. Computer Vision—ECCV 2016.

[41] Cox D.R. (1958). The Regression Analysis of Binary Sequences. J. R. Stat. Soc. Ser. B Methodol..

[42] Sabil A., Glos M., Günther A., Schöbel C., Veauthier C., Fietze I., Penzel T. (2019). Comparison of apnea detection using oronasal thermal airflow sensor, nasal pressure transducer, respiratory inductance plethysmography and tracheal sound sensor. J. Clin. Sleep Med..

[43] Holm B., Borsky M., Arnardottir E.S., Serwatko M., Mallett J., Islind A.S., Óskarsdóttir M. (2024). BreathFinder: A method for non-invasive isolation of respiratory cycles utilizing the thoracic respiratory inductance plethysmography signal. Nat. Sci. Sleep.

[44] Remmers J.E., Degroot W.J., Sauerland E.K., Anch A.M. (1978). Pathogenesis of upper airway occlusion during sleep. J. Appl. Physiol. Respir. Environ. Exerc. Physiol..

[45] McNicholas W.T., Korkalainen H. (2023). Translation of obstructive sleep apnea pathophysiology and phenotypes to personalized treatment: A narrative review. Front. Neurol..

[46] Schwartz A.R., Barnes M., Hillman D., Malhotra A., Kezirian E., Smith P.L., Hoegh T., Parrish D., Eastwood P.R. (2012). Acute upper airway responses to hypoglossal nerve stimulation during sleep in obstructive sleep apnea. Am. J. Respir. Crit. Care Med..

[47] Eastwood P.R., Barnes M., Walsh J.H., Maddison K.J., Hee G., Schwartz A.R., Smith P.L., Malhotra A., McEvoy R.D., Wheatley J.R. (2011). Treating obstructive sleep apnea with hypoglossal nerve stimulation. Sleep.

[48] Bai S., Kolter J.Z., Koltun V. (2018). An empirical evaluation of generic convolutional and recurrent networks for sequence modeling. arXiv.

[49] He K., Zhang X., Ren S., Sun J. Deep residual learning for image recognition. Proceedings of the IEEE Conference on Computer Vision and Pattern Recognition (CVPR).

[50] Vaswani A., Shazeer N., Parmar N., Uszkoreit J., Jones L., Gomez A.N., Kaiser Ł., Polosukhin I. (2017). Attention is all you need. Advances in Neural Information Processing Systems 30.

[51] Gulati A., Qin J., Chiu C.-C., Parmar N., Zhang Y., Yu J., Han W., Wang S., Zhang Z., Wu Y. Conformer: Convolution-augmented Transformer for speech recognition. Proceedings of the Interspeech 2020.

[52] Lee-Thorp J., Ainslie J., Eckstein I., Ontañón S. (2022). FNet: Mixing tokens with Fourier transforms. Proceedings of the 2022 Conference of the North American Chapter of the Association for Computational Linguistics: Human Language Technologies.

[53] Ronneberger O., Fischer P., Brox T., Navab N., Hornegger J., Wells W.M., Frangi A.F. (2015). U-Net: Convolutional networks for biomedical image segmentation. Medical Image Computing and Computer-Assisted Intervention—MICCAI 2015.

[54] Hu J., Shen L., Sun G. Squeeze-and-Excitation networks. Proceedings of the IEEE Conference on Computer Vision and Pattern Recognition (CVPR).

