# Supplementary material for: Triaxial Accelerometry Sensor for Respiratory-Effort Phase Landmark Detection During Sleep: A Cross-Modal Weak Supervision Approach
Source: Bioengineering (Basel). 2026 Oct 16;13(10):1205. doi: 10.3390/bioengineering13101205 (PMC13701435; doi:10.3390/bioengineering13101205)
Supplement: Supplementary file 1 [file bioengineering-13-01205-s001.zip › bioengineering-4589856-supplementary.pdf]

Supplementary Material

Supplementary Note S1. Expert Validation of the RIP-Trough-Derived Operational Phase Landmarks

1. Materials and Methods

For each participant, the analyzable full-night PSG recording was divided into non-overlapping 60 s epochs, from which 10 epochs were selected without replacement using computer-generated random sampling. This subject-stratified procedure yielded 100 epochs from 10 participants (100 min in total). Sampling was completed before expert review and was independent of the automatically detected RIP troughs and model predictions.

Two sleep experts annotated respiratory onsets in all 100 epochs. Both experts reviewed the synchronized nasal-pressure, oronasal thermal-airflow, and thoracoabdominal RIP signals. Respiratory onset was defined as the first discernible inspiratory deflection from the end-expiratory baseline in the airflow signals. Events that could not be reliably timed were marked as unscorable. Both experts were blinded to the TAS signals, model outputs, automatically detected RIP troughs, Gaussian weak-label targets, and each other's annotations.

Inter-rater agreement was evaluated across all 100 epochs before adjudication. Annotations were matched one-to-one within ± 0.50 s, and agreement was summarized using event-level positive agreement and the temporal differences between matched annotations. Disagreements were subsequently reviewed jointly, and consensus annotations were incorporated into the final expert reference. The RIP trough-extraction algorithm and its preprocessing parameters were frozen before annotation. Automatic troughs were matched one-to-one to the final expert annotations within prespecified temporal tolerances of ± 0.50 and ± 1.00 s.

2. Results

After adjudication, the final consensus reference contained 1428 respiratory-onset annotations across the 100 sampled epochs, of which 1377 were matched within ± 0.50 s. Event-level positive agreement was 0.964. For the matched annotations, the median absolute inter-rater difference was 0.031 s (IQR, 0.000–0.094 s), and the mean difference between expert 2 and expert 1 was −0.006 s, with 95% limits of agreement from −0.235 to 0.222 s.

The median absolute temporal difference between the expert annotations and the frozen automatic RIP troughs was 0.125 s (IQR, 0.062–0.250 s); 76.8% and 94.0% of expert-annotated onsets were within ± 0.25 and ± 0.50 s of an automatic trough, respectively. Event-level F1 scores were 0.873 at a ± 0.50 s matching tolerance and 0.896 at a ± 1.00 s tolerance. In the polarity analysis, 96.4% of expert annotations were temporally closer to a trough than to a peak of the processed RIP signal. These findings support the RIP trough as an expert-validated operational proxy for respiratory onset under the recorded polarity and specified preprocessing procedure, although it should not be interpreted as an error-free physiological gold standard.

Tables

Table S1. Model sources, architecture specifications, and output adaptations.

Model	Parameters	Model family/source	Core architecture	Sequence-output adaptation
CNN-BiLSTM	3,103,745	LSTM family	Three-level convolutional encoder; one-layer bidirectional LSTM with 416 units per direction	Transposed-convolution decoder
TCN	3,043,665	Bai et al. [48]	Seven residual blocks; width 208; kernel 5; dilations 1–64	Dense one-dimensional output
ResNet1D	3,248,201	He et al. [49]	Width 200; eight residual kernel-5 blocks; dilations 1–128	Dense one-dimensional output
Dilated Conv-GRU	3,110,433	GRU family	Convolutional encoder; dilated residual blocks; bidirectional GRU with 440 units per direction	Symmetric temporal decoder
Transformer	3,136,417	Vaswani et al. [50]	Dimension 224; seven layers; eight heads; feed-forward dimension 512	Interpolation and convolutional output
Parameter-matched Conformer	3,222,273	Gulati et al. [51]	Dimension 160; eight blocks; eight heads; feed-forward dimension 320; convolution kernel 31	Interpolation and convolutional output
Fourier Mixer	3,285,697	FNet family; Lee-Thorp et al. [52]	Dimension 256; eight Fourier mixing blocks; feed-forward dimension 768	Dense one-dimensional output
SE-U-Net	3,121,217	Ronneberger et al. [53]; Hu et al. [54]	Residual U-Net with channels 32–512 and four encoder-decoder levels	One-dimensional skip decoder
Multiscale CWT Conformer	3,362,315	This study; Conformer family	Post-standardization channel recalibration; 64-channel high-resolution branch; eight 160-dimensional Conformer blocks	High-resolution feature fusion

Single models accepted a 27 × 1024 TAS-derived input and produced 1024 pointwise RIP-derived landmark logits; the proposed method fused reconstructed logits from two component models and the standard and shifted grids.

Table S2. Fold-level outer-test performance and training-side validation-only operating-point selection for multi-offset dual-Conformer fusion.

Outer test	Fusion F1	CWT-Conformer F1	Matched-Conformer F1	Delta vs CWT	Delta vs matched	CWT weight	Matched weight	Threshold	Inner validation
A	0.9552	0.9526	0.8784	+0.0026	+0.0767	0.70	0.30	0.15	B
B	0.7215	0.7058	0.7235	+0.0157	-0.0021	0.30	0.70	0.20	C
C	0.7511	0.7061	0.7407	+0.0450	+0.0104	0.40	0.60	0.25	D
D	0.7481	0.7304	0.7334	+0.0177	+0.0147	0.40	0.60	0.30	E
E	0.7399	0.7105	0.7121	+0.0293	+0.0278	0.50	0.50	0.20	F
F	0.8805	0.8664	0.8698	+0.0141	+0.0107	0.20	0.80	0.25	G
G	0.8192	0.7504	0.8154	+0.0688	+0.0038	0.20	0.80	0.20	H
H	0.7178	0.7389	0.6944	-0.0211	+0.0234	0.40	0.60	0.15	I
I	0.8012	0.7952	0.7346	+0.0060	+0.0666	0.70	0.30	0.30	J
J	0.8442	0.8236	0.8347	+0.0206	+0.0095	0.50	0.50	0.20	A
Mean ± SD	0.7979 ± 0.0778	0.7780 ± 0.0818	0.7737 ± 0.0687	+0.0199 [0.0063, 0.0347]; 9/10	+0.0241 [0.0101, 0.0413]; 9/10	0.43 ± 0.18	0.57 ± 0.18	0.22 ± 0.05	One per fold

F1 uses one-to-one event matching within ± 1.0 s. Delta columns are multi-offset fusion minus the indicated component. Model weights sum to one and, together with the event threshold, were selected only on the listed inner-validation participant before outer-test access. The final row gives participant mean ± SD; confidence intervals are paired participant bootstrap intervals.

Table S3. Descriptive landmark agreement across sleep stages and body positions during scored sleep for the Multiscale CWT component.

Stage	n_subjects*	Duration (min)	RIP landmarks	TP	FP	FN	Precision	Recall	Pooled F1	Accuracy	Subject-level F1
**Sleep stage**											
N1	8 (8)	234.7	3385	2446	988	939	0.71	0.72	0.72	0.83	0.68 ± 0.09
N2	8 (8)	2111.9	30,112	25,695	5501	4417	0.82	0.85	0.84	0.84	0.84 ± 0.09
N3	6 (6)	434.0	6369	5882	1134	487	0.84	0.92	0.88	0.86	0.82 ± 0.11
REM	8 (8)	697.6	9734	7911	2668	1823	0.75	0.81	0.78	0.83	0.80 ± 0.12
Wake	7 (7)	615.0	9078	6013	2702	3065	0.69	0.66	0.68	0.81	0.67 ± 0.11

Stage	n_subjects*	Duration (min)	RIP landmarks	TP	FP	FN	Precision	Recall	Pooled F1	Accuracy	Subject-level F1
**Position**											
Supine	8 (8)	2496.7	35,245	30,091	6657	5154	0.82	0.85	0.84	0.85	0.82 ± 0.09
Right lateral	8 (8)	772.5	10,764	8875	3016	1889	0.75	0.82	0.78	0.81	0.80 ± 0.09
Left lateral	3 (2)	207.6	3577	2963	606	614	0.83	0.83	0.83	0.78	0.86 ± 0.12

*Values are n_subjects with valid annotated duration (n_subjects with at least one reference event). C and D were excluded because their continuous sleep-stage files were unscored. REM, rapid eye movement; TP, true positive; FP, false positive; FN, false negative.

Table S4. Sampled strict-streaming replay of the Multiscale CWT Conformer component.
Values are mean ± SD across participants unless noted. Replay used only the Multiscale CWT component,

Mode	Precision	Recall	F1	Micro F1	Median response delay (s)	95th-percentile delay (s)	Mean real-time factor
Zero additional look-ahead	0.76 ± 0.17	0.80 ± 0.16	0.77 ± 0.16	0.78	0.686	1.508	0.00398
One-second look-ahead	0.75 ± 0.16	0.83 ± 0.12	0.78 ± 0.13	0.79	1.744	2.509	0.00398

three sampled 5-min blocks per participant, a trailing 32 s window, 1 s updates, final-second outputs, and validation-selected thresholds. It did not evaluate the multi-offset fusion and was not full-night or target-hardware validation.

Figures

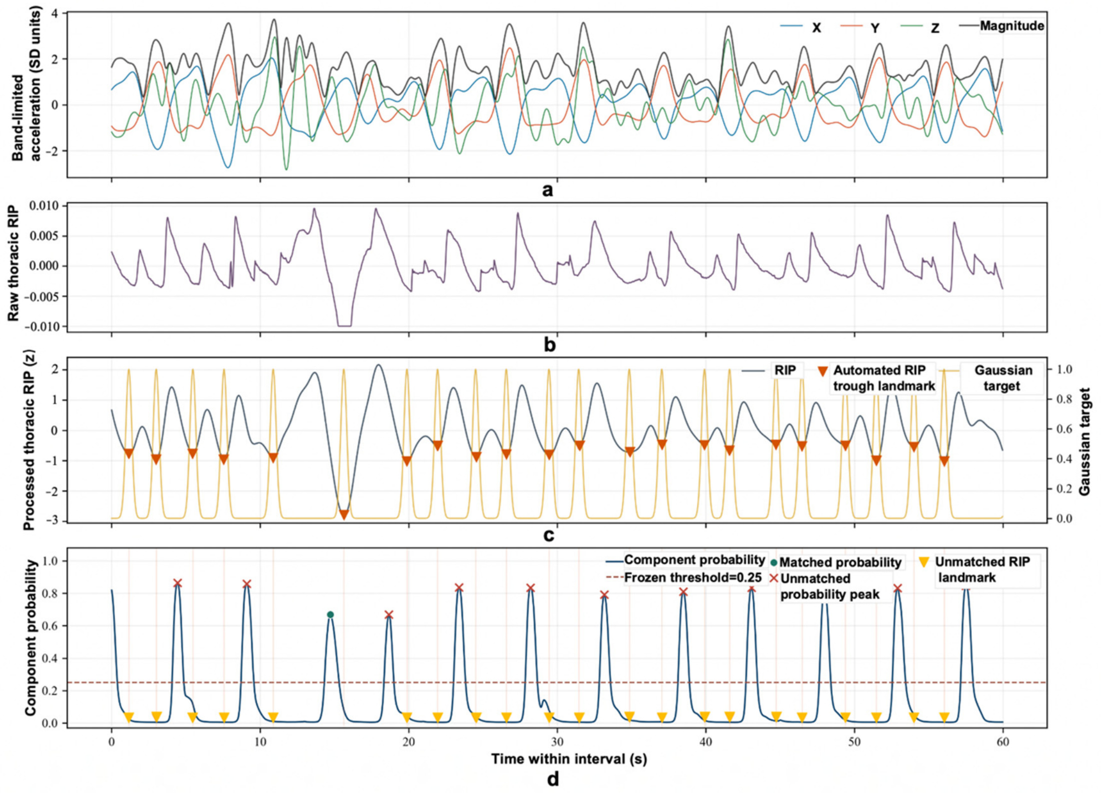

Supplementary Figure S1. Representative false-negative-dominant interval for the Multiscale CWT Conformer. A 60-s standard-grid outer-test interval from Participant D is shown. (a) Band-limited triaxial acceleration signals and vector magnitude. (b) Raw thoracic respiratory inductance plethysmography (RIP). (c) Processed thoracic RIP with RIP-trough-derived operational phase landmarks and Gaussian targets. (d) Component probability, the frozen fold-specific threshold, matched probability peaks, unmatched probability peaks, and unmatched RIP landmarks. Weak and irregular respiratory modulation in the TAS signals coincided with numerous missed landmarks (TP = 1, FP = 11, FN = 21; F1 = 0.06).

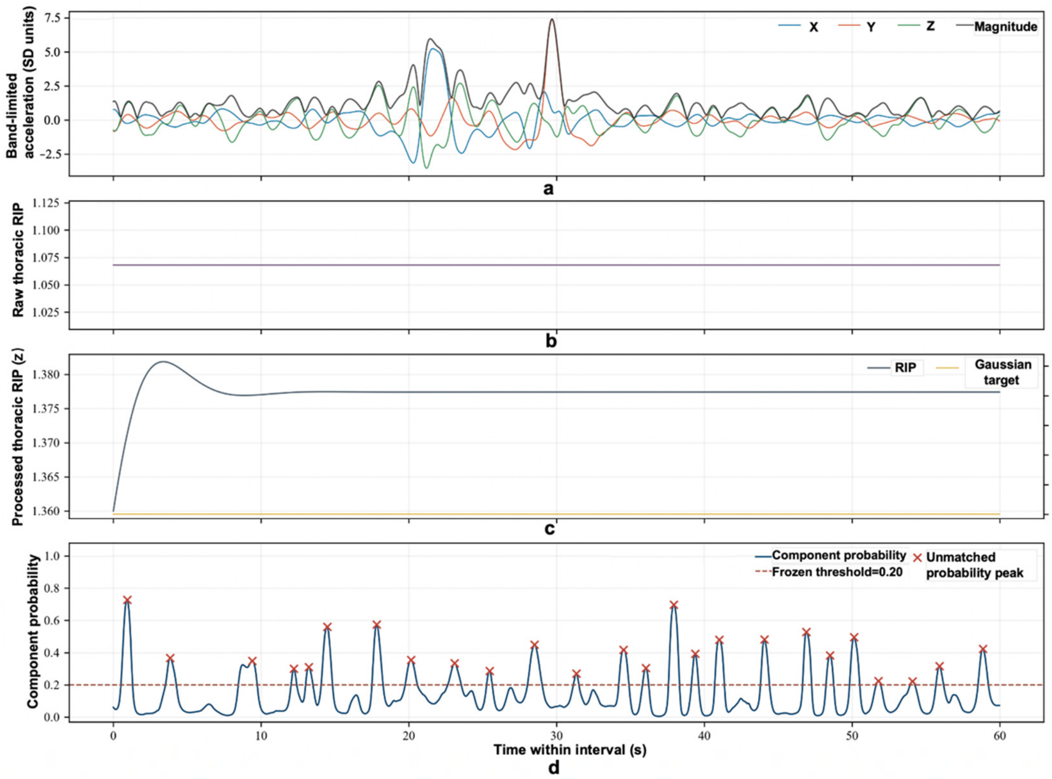

Supplementary Figure S2. Representative interval with unmatched predictions in the absence of RIP-derived landmarks. A 60-s standard-grid outer-test interval from Participant E is shown. (a) Band-limited triaxial acceleration signals and vector magnitude, including transient multiaxial activity. (b) Raw thoracic respiratory inductance plethysmography (RIP), which remained nearly constant throughout the interval. (c) Processed thoracic RIP and Gaussian target sequence; no RIP-trough-derived operational phase landmarks were generated. (d) Component probability, the frozen fold-specific threshold, and unmatched probability peaks. Twenty-five probability peaks crossed the threshold, but none could be matched because the RIP-derived reference contained no landmarks (TP = 0, FP = 25, FN = 0).

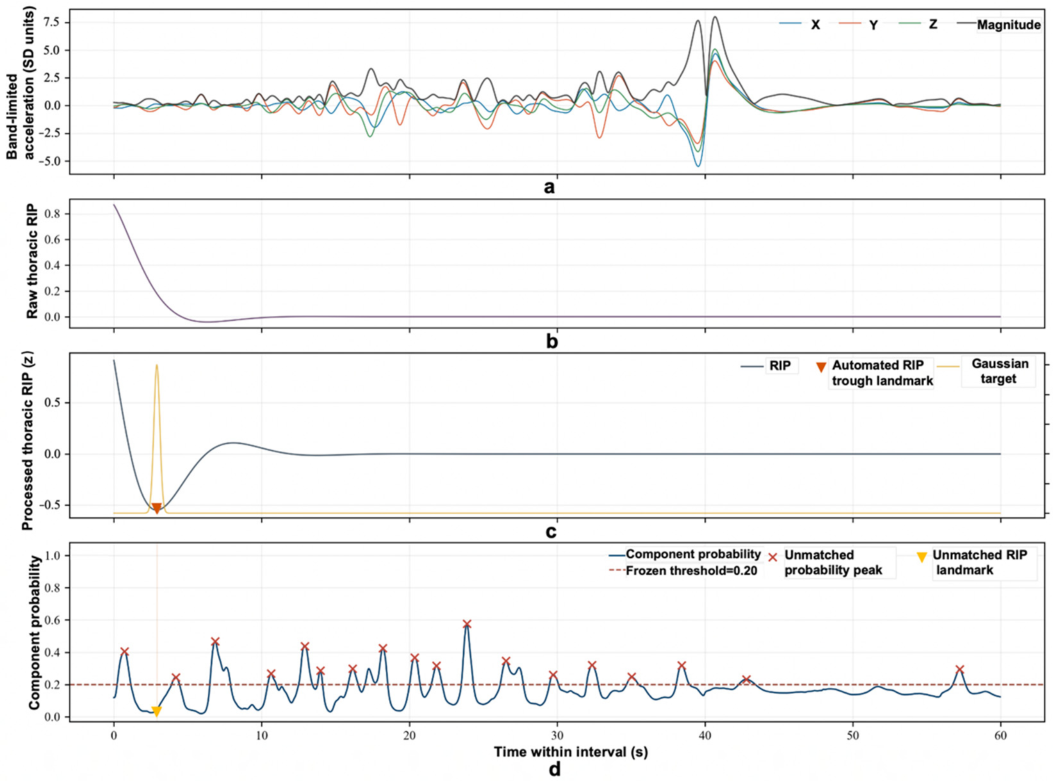

Supplementary Figure S3. Representative disagreement interval during irregular and attenuated thoracic RIP morphology. A 60-s standard-grid outer-test interval from Participant C is shown. (a) Band-limited triaxial acceleration signals and vector magnitude, including a prominent transient multiaxial deflection. (b) Raw thoracic respiratory inductance plethysmography (RIP), which rapidly attenuated toward a near-constant baseline. (c) Processed thoracic RIP with one RIP-trough-derived operational phase landmark and its Gaussian target. (d) Component probability, the frozen fold-specific threshold, unmatched probability peaks, and the unmatched RIP landmark. No model detections were matched to the reference landmark (TP = 0, FP = 18, FN = 1; F1 = 0.00).

Reference

48. Bai, S.; Kolter, J.Z.; Koltun, V. An empirical evaluation of generic convolutional and recurrent networks for sequence modeling. *arXiv* 2018, *arXiv:*1803.01271. https://doi.org/10.48550/arXiv.1803.01271.
49. He, K.; Zhang, X.; Ren, S.; Sun, J. Deep residual learning for image recognition. In Proceedings of the IEEE Conference on Computer Vision and Pattern Recognition (CVPR), Las Vegas, NV, USA, 27–30 June **2016**; pp. 770–778. https://doi.org/10.1109/CVPR.2016.90.
50. Vaswani, A.; Shazeer, N.; Parmar, N.; Uszkoreit, J.; Jones, L.; Gomez, A.N.; Kaiser, Ł.; Polosukhin, I. Attention is all you need. In Advances in Neural Information Processing Systems 30; Curran Associates, Inc.: Red Hook, NY, USA, **2017**; pp. 5998–6008.
51. Gulati, A.; Qin, J.; Chiu, C.-C.; Parmar, N.; Zhang, Y.; Yu, J.; Han, W.; Wang, S.; Zhang, Z.; Wu, Y.; Pang, R. Conformer: Convolution-augmented Transformer for speech recognition. In Proceedings of Interspeech 2020, Shanghai, China, 25–29 October 2020; pp. 5036–5040. https://doi.org/10.21437/Interspeech.2020-3015.
52. Lee-Thorp, J.; Ainslie, J.; Eckstein, I.; Ontañón, S. FNet: Mixing tokens with Fourier transforms. In Proceedings of the 2022 Conference of the North American Chapter of the Association for Computational Linguistics: Human Language Technologies, Seattle, WA, USA, 10–15 July 2022; Association for Computational Linguistics: Stroudsburg, PA, USA, **2022**; pp. 4296–4313. https://doi.org/10.18653/v1/2022.naacl-main.319.
53. Ronneberger, O.; Fischer, P.; Brox, T. U-Net: Convolutional networks for biomedical image segmentation. In Medical Image Computing and Computer-Assisted Intervention—

MICCAI 2015; Navab, N.; Hornegger, J.; Wells, W.M.; Frangi, A.F., Eds.; Lecture Notes in Computer Science; Springer: Cham, Switzerland, 2015; Volume 9351, pp. 234–241. https://doi.org/10.1007/978-3-319-24574-4_28.
54. Hu, J.; Shen, L.; Sun, G. Squeeze-and-Excitation networks. In Proceedings of the IEEE Conference on Computer Vision and Pattern Recognition (CVPR), Salt Lake City, UT, USA, 18–23 June 2018; pp. 7132–7141. https://doi.org/10.1109/CVPR.2018.00745.
